# Phylogenomics of *Salvia* L. subgenus *Calosphace* (Lamiaceae)

**DOI:** 10.3389/fpls.2021.725900

**Published:** 2021-10-15

**Authors:** Sabina Irene Lara-Cabrera, Maria de la Luz Perez-Garcia, Carlos Alonso Maya-Lastra, Juan Carlos Montero-Castro, Grant T. Godden, Angelica Cibrian-Jaramillo, Amanda E. Fisher, J. Mark Porter

**Affiliations:** ^1^Laboratorio de Sistemática Molecular de Plantas, Facultad de Biología, Universidad Michoacana de San Nicolás de Hidalgo, Morelia, Mexico; ^2^Departamento de Botánica y Zoología, Centro Universitario de Ciencias Biológicas y Agropecuarias, Universidad de Guadalajara, Guadalajara, Mexico; ^3^Department of Ecology, Evolution, and Environmental Biology, Columbia University, New York, NY, United States; ^4^Florida Museum of Natural History, University of Florida, Gainesville, FL, United States; ^5^Laboratorio Nacional de Genómica para la Biodiversidad, Unidad de Genómica Avanzada del Centro de Investigación y de Estudios Avanzados del instituto Politécnico Nacional, Irapuato, Mexico; ^6^Department of Biological Sciences, California State University, Long Beach, CA, United States; ^7^California Botanic Garden, Claremont, CA, United States

**Keywords:** Hyb-Seq, chloroplast, section, nuclear, monophyly

## Abstract

The evolutionary relationships of *Salvia* have been difficult to estimate. In this study, we used the Next Generation Sequencing method Hyb-Seq to evaluate relationships among 90 Lamiaceae samples, including representatives of *Mentheae, Ocimeae, Salvia* subgenera *Audibertia, Leonia, Salvia*, and 69 species of subgenus *Calosphace*, representing 32 of Epling's sections. A bait set was designed in MarkerMiner using available transcriptome data to enrich 119 variable nuclear loci. Nuclear and chloroplast loci were assembled with *hybphylomaker* (HPM), followed by coalescent approach analyses for nuclear data (ASTRAL, BEAST) and a concatenated Maximum Likelihood analysis of chloroplast loci. The HPM assembly had an average of 1,314,368 mapped reads for the sample and 527 putative exons. Phylogenetic inferences resolved strongly supported relationships for the deep-level nodes, agreeing with previous hypotheses which assumed that subgenus *Audibertia* is sister to subgenus *Calosphace*. Within subgenus *Calosphace*, we recovered eight monophyletic sections *sensu* Epling, *Cardinalis, Hastatae, Incarnatae*, and *Uricae* in all the analyses (nDNA and cpDNA), *Biflorae, Lavanduloideae*, and *Sigmoideae* in nuclear analyses (ASTRAL, BEAST) and *Curtiflorae* in ASTRAL trees. Network analysis supports deep node relationships, some of the main clades, and recovers reticulation within the core *Calosphace*. The chloroplast phylogeny resolved deep nodes and four monophyletic *Calosphace* sections. Placement of *S. axillaris* is distinct in nuclear evidence and chloroplast, as sister to the rest of the *S*. subg. *Calosphace* in chloroplast and a clade with “*Hastatae* clade” sister to the rest of the subgenus in nuclear evidence. We also tested the monophyly of *S. hispanica, S. polystachia, S. purpurea*, and *S*. *tiliifolia*, including two samples of each, and found that *S. hispanica* and *S. purpurea* are monophyletic. Our baits can be used in future studies of Lamiaceae phylogeny to estimate relationships between genera and among species. In this study, we presented a Hyb-Seq phylogeny for complex, recently diverged *Salvia*, which could be implemented in other Lamiaceae.

## Introduction

Phylogenetic relationships for many plant groups have been studied through the last 30–40 years at deep (APG, 1998; Zeng et al., 2017; Breinholt et al., 2021) and shallow phylogenetic levels (Wells et al., 2020), mostly through Sanger sequencing (Sanger et al., 1977) and recently through Next Generation Sequencing (Wanke et al., 2017; Carlsen et al., 2018; Herrando-Moraira and The Cardueae Radiations Group, 2018; Villaverde et al., 2018; Carter et al., 2019; Johnson et al., 2019). However, in groups with recent radiation events (Larridon et al., 2020) such as *Salvia* L. (Walker and Sytsma, 2007; Jenks et al., 2013; Fragoso-Martínez et al., 2018; González-Gallegos et al., in press), many questions remain at the shallow-phylogenetic scale, such as relationships among sections, among species, and species monophyly.

The sages (*Salvia*) with *ca*. 1,000 species (Harley et al., 2004; Drew et al., 2017), are among the largest angiosperm genera (Frodin, 2004). They are widely distributed with many economically important species (Wu et al., 2012; Lopresti, 2017). *Salvia* flowers are bilabiate and have evolved a wide variety of showy colors and shapes (Lara-Cabrera et al., in press), as well as staminal levers and other morphological adaptations to pollinators (Claßen-Bockhoff et al., 2004; Wester and Claßen-Bockhoff, 2011; Benítez-Vieyra et al., 2014; Kriebel et al., 2019, 2020; Celep et al., 2020). Previous *Salvia* phylogenies that employed few, e.g., <5–10, chloroplast or nuclear coding and non-coding loci were successful in reconstructing relationships at many deep-level nodes. These studies showed that *Salvia* is polyphyletic with five embedded genera, namely, *Dorystaechas* Boiss. and Heldr. ex Benth., *Meriandra* Benth., *Perovskia* Kar., *Rosmarinus* L., and *Zhumeria* Rech. f. and Wendelbo (Walker et al., 2004; Walker, 2006; Walker and Sytsma, 2007). *Salvia* species are classified into five subgenera, namely, *Salvia, Audibertia* J. B. Walker, B. T. Drew and K. J. Sytsma, *Calosphace* (Benth.) Epling, *Leonia* Cerv., and *Sclarea* Mill. A proposal to “lump” these genera into *Salvia* would add five more subgenera to *Salvia* (Drew et al., 2017), which are *Dorystaechas* (Boiss. and Heldr. ex Benth.) J. B.Walker, B. T. Drew, and J. G. González, *Meriandra* (Benth.); J. B. Walker, B. T. Drew, and J. G. González, *Perovskia* (Kar.); J. B. Walker, B. T. Drew, and J. G. González, *Rosmarinus* (L.); J. B. Walker, B. T. Drew, and J. G. González, and *Zhumeria* (Rech.f. and Wendelbo); J. B. Walker, B. T. Drew, and J. G. González. Among these, we focused in this study mainly on the American subgenus *Calosphace* and some representatives in subgenera *Audibertia, Leonia*, and *Salvia s.s*.

*Salvia* subg. *Calosphace* is distributed from southern USA to Argentina (Ramamoorthy and Elliott, 1998; Walker et al., 2004), with *ca*. 580 (González-Gallegos et al., in press) to 600 species (Martínez-Gordillo et al., 2017). It is most diverse in Mexico and Central America (275 species), the Andes (155 species), Eastern South America (60 species), and the Antilles (45 species; Jenks et al., 2013). Given *S*. subg. *Calosphace* species diversity and morphological complexities, it has been classified into 102 sections (Epling, 1939, 1940, 1941, 1944, 1947, 1951; Epling and Mathias, 1957; Epling and Jativa, 1963). However, the sectional classification has been criticized (Standley and Williams, 1973; Torke, 2000; Walker, 2006; Wood, 2007), given the few characters employed to define sections, and disjunct distribution of some species. Regardless, Epling's classification is recognized as a necessary starting point to further the study on *Salvia* until a new monograph is compiled (Ramamoorthy, 1984; Wood, 2007; Klitgaard, 2012).

Previous phylogenetic studies of *Calosphace* resolved *S*. *axillaris* Moc. and Sessé sister to the rest of the subgenus (Walker et al., 2004; Walker and Sytsma, 2007; Jenks et al., 2013; Drew et al., 2017; Fragoso-Martínez et al., 2018; Kriebel et al., 2019), followed by the *Hastatae* clade (*Salvia patens* Ort. + *Salvia vitifolia* Benth.); members of the *S*. sects. *Tomentellae, Dusenostachys, Uliginosae, Erytrostachys, Micranthae, Fulgentes*, and *Membranaceae* (Fragoso-Martínez et al., 2018) or *Fulgentes* was paraphyletic to members of sects. *Cardinalis* and *Flocculosae* (Jenks et al., 2013). The “Core *Calosphace*” contains the most species and relationships within this clade that have been difficult to resolve or have had low branch support. The “Core *Calosphace*” clade was initially described by Walker (2006) and refers to a clade of “core radiation” that is “difficult to characterize morphologically but is well-supported in the molecular analyses...”. It has been hypothesized that recent divergence events are clouding the phylogenetic signal, which could be further tested with expanded taxon sampling and additional phylogenetically informative sequence data (Olvera-Mendoza et al., 2020; Villaverde et al., 2020). This was attempted by Fragoso-Martínez et al. (2017) and Kriebel et al. (2019) using hybrid enrichment protocols across *Salvia* and to test sectional monophyly of the *Calosphace*. The Anchored Hybrid Enrichment (AHE) Angiosperm kit v. 1 (Buddenhagen et al., 2016) was tested on 12 *Salvia* species and captured 399 nuclear loci (Fragoso-Martínez et al., 2017) and later the protocol was used for 35 *Salvia* (13 *Calosphace* and 2 *Audibertia*) species capturing 316 nuclear genes (Kriebel et al., 2019). Both phylogenies improved clade resolution as compared to previous sequencing studies (Walker et al., 2004; Walker and Sytsma, 2007; Jenks et al., 2013; Will and Claßen-Bockhoff, 2017; Fragoso-Martínez et al., 2018; Hu et al., 2018).

In this study, we used the Hyb-Seq protocol (Weitemier et al., 2014) for target enrichment of low copy nuclear exons and flanking regions and genome skimming of organellar genomes. Hyb-Seq has been successfully used to solve shallow-level phylogenetic relationships in *Asclepias* L. (Straub et al., 2011, 2012), Annonaceae (Couvreur et al., 2019), *Asteraceae* (Mandel et al., 2017; Herrando-Moraira and The Cardueae Radiations Group, 2019; Johnson et al., 2019; Jones et al., 2019), Poaceae (Fisher et al., 2016), and *Rubus* (Carter et al., 2019), among others. We used MarkerMiner (Chamala et al., 2015) to identify low copy nuclear loci in 22 Lamiaceae transcriptomes (including *Salvia officinalis* L. and *S. splendens* Sellow ex Schult.) and design both general and specific purpose bait sets. We sampled a total of 90 Lamiaceae from tribes Mentheae and Ocimeae, 75 samples represent 32 of Epling's *S*. subg. *Calosphace* sections. Our goals were to test classification of Epling and relationships found in previous studies of subg. *Calosphace*; test species monophyly for four important and morphologically complex species. Furthermore, we aimed to identify sufficiently polymorphic loci for future studies in *Salvia*.

## Methods

### Taxonomic Sampling

The study materials consisted of 90 *Lamiaceae* from nine genera which were sampled (Supplementary Table 1). Exactly 10 species were sampled from tribe *Mentheae* [*Agastache pallidiflora* subsp. *neomexicana* (Briq.) Lint and Epling, *Dracocephalum parviflorum* Nutt., *Hedeoma drummondii* Benth., *Lepechinia hastata* (A. Gray) Epling, *Lepechinia* sp*., Lycopus americanus* Muhl., *Melissa officinalis* L., *Poliomintha incana* (Torr.) A. Gray, and *Prunella vulgaris* L.] and one species was sampled from the tribe *Ocimeae* [*Cantinoa mutabilis* (Rich.) Harley and J. F. B. Pastore] to root the trees (Li et al., 2016).

Multiple subgenera of *Salvia* were represented in our sampling, two each from the *S*. subg. *Audibertia* sect. *Audibertia* (*S. brandegeei* Munz and *S. sonomensis* Greene) and *S*. subg. *Salvia* sect. *Salvia* (the Mediterranean *S. officinalis* and the Malagasy *S. sessilifolia* A. Gray ex S. Watson), and one from the *S*. subg. *Leonia* sect. *Salviastrum* [S. *texana* (Scheele) Torr.]. From the *S*. subgenus *Calosphace*, we sampled 72 species (Supplementary Table 1) in all, representing 32 of the 102 sections *sensu* Epling. Our sampling represents the geographic range of the taxon in Mexico (67 species; Supplementary Table 1) and includes five additional species from Central and South America (*S. pauciserrata* Benth., *S. scutellarioides* Kunth, *S. splendens, S. squalens* Kunth, and *S. tubiflora* Sm.). Seven species were sampled for molecular study for the first time (*S. brachyodonta* Briq., *S. decora* Epling, *S. dichlamys* Epling, *S. perblanda* Epling, *S. puberula* Fernald, *S. purepecha* Bedolla, S. Lara Cabrera and Zamudio, and *S. roscida* Fernald). Additionally, we included two samples from distinct provenances for *Salvia hispanica* L., *Salvia polystachia* Cav., *Salvia purpurea* Cav., and *Salvia tiliifolia* Vahl., to assess their monophyly, which further tested the resolving power of this protocol.

### Phylogenetic Marker Selection, Bait Design, and DNA Sequencing

Genomic DNA was isolated from 10 mg of silica-dried leaf material using a modified 2X CTAB protocol (Doyle and Doyle, 1987). DNAs were quantified using a Qubit 2.0 fluorometer (Thermo Fisher Scientific Inc., Waltham, MA, USA) and diluted to a concentration of 20 ng/μl. Afterward, 60 μl of DNA solution were transferred to a 96-well plate and shipped to Rapid Genomics (Gainesville, FL, USA) for library preparation, hybrid enrichment of nuclear loci, and paired-end (2 × 150 bp) sequencing on the Illumina HiSeq 2500 instrument (Thermo Fisher Scientific Inc.).

A multipurpose bait set was designed for use across independent research projects with *Salvia*, Acanthaceae, Clusiaceae, Lamiales, and Polemoniaceae. To select loci and provide sequence data for bait design for the *Salvia* and Lamiales studies, we analyzed a set of 77 transcriptomes from the One Thousand Plant Transcriptomes Initiative (OneKp), including 68 from Lamiales and 9 from outgroup taxa representing Boraginales, Gentianales, and Solanales (One Thousand Plant Transcriptomes Intitiative, 2019), and an additional transcriptome for *S. splendens* Sellow ex Wied-Neuw. in Genbank [Ge et al., 2014; https://www.ncbi.nlm.nih.gov/bioproject/422035 (Taxonomy ID: 180675)]. We used the MarkerMiner 1.0 (Chamala et al., 2015) pipeline with its default settings to assess putative orthology among transcripts in our data set with a set of *Arabidopsis thaliana* (L.) Heynh. transcripts from genes that were identified as single- (or low-)copy across angiosperms by an orthology analysis of 20 genomes (De Smet et al., 2013), mapping to chromosomes 1, 2, 3, 4, and 5 in the *A. thaliana* genome (Table 1); at the time we had no fully annotated Lamiaceae genome. Gene clusters identified by MarkerMiner were aligned with MAFFT (Katoh et al., 2002) and individually reviewed for marker selection.

**Table 1:** HPM assembly characteristics per sample for 90 samples targeting 119 nuclear genes, 26 genes were later filtered through the next steps in HPM.

**Species**	**Total nr. reads**	**Nr. paired reads**	**Nr. forward unpaired reads**	**Nr. reverse unpaired reads**	**Nr. mapped reads**	**% Mapped reads**
*Agastache sp*.	4,795,650	2,341,728	68,327	41,718	1,328,625	27.70
*Dracocephalum parviflorum*	3,520,197	1,720,163	48,401	30,827	1,314,368	37.34
*Hedeoma drummondii*	3,279,317	1,590,347	63,879	34,020	1,099,519	33.53
*Cantinoa mutabilis*	2,132,204	1,028,383	50,662	24,360	606,056	28.42
*Lepechinia hastata*	1,769,299	859,751	31,856	17,489	343,698	19.43
*Lepechinia sp*.	2,195,750	1,071,781	32,194	19,383	589,054	26.83
*Lycopus americanus*	4,277,985	2,070,253	92,390	42,301	1,518,980	35.51
*Melissa officinalis*	3,152,455	1,540,899	44,739	25,608	898,696	28.51
*Poliomintha incana*	3,122,237	1,521,007	47,608	32,011	646,254	20.70
*Prunella vulgaris*	1,390,762	676,453	24,947	12,281	346,864	24.94
*Salvia aequidistans*	3,916,050	1,893,614	89,280	38,855	1,372,874	35.06
*Salvia amarissima*	2,304,666	1,110,067	51,892	32,269	745,462	32.35
*Salvia areolata*	4,393,342	2,139,596	66,078	46,336	1,772,640	40.35
*Salvia axillaris*	2,181,941	1,049,876	45,833	35,955	636,302	29.16
*Salvia azurea*	4,533,709	2,206,512	70,640	48,461	1,751,642	38.64
*Salvia blepharophylla*	4,815,199	2,345,302	74,133	47,050	2,001,549	41.57
*Salvia brachyodonta*	4,295,551	2,082,412	83,182	46,634	1,559,488	36.30
*Salvia brandegeei*	2,312,395	1,125,113	39,632	21,346	921,779	39.86
*Salvia breviflora*	853,293	409,465	19,377	14,703	**255,228**	29.91
*Salvia cacaliifolia*	3,228,987	1,571,229	55,214	30,241	1,027,027	31.81
*Salvia chamaedryoides*	4,695,712	2,269,098	127,848	28,614	1,911,882	40.72
*Salvia chiapensis*	2,079,209	1,002,758	46,707	26,286	648,845	31.21
*Salvia cinnabarina*	3,864,253	1,800,499	240,431	20,724	1,098,123	28.42
*Salvia clinopodioides*	2,663,514	1,230,419	186,821	13,104	640,127	24.03
*Salvia coahuilensis*	6,760,892	3,139,087	448,389	30,673	2,648,399	39.17
*Salvia connivens*	3,955,980	1,837,482	257,520	21,352	1,614,082	40.80
*Salvia curtiflora*	4,897,789	2,272,279	326,202	23,555	2,001,741	40.87
*Salvia curviflora*	1,480,065	654,763	158,472	11,637	414,403	28.00
*Salvia decora*	2,188,709	941,220	291,136	14,110	723,442	33.05
*Salvia dichlamys*	4,118,055	1,911,522	270,852	21,762	1,667,989	40.50
*Salvia disjuncta*	3,907,516	1,790,553	304,076	18,348	1,614,927	41.33
*Salvia divinorum*	3,160,115	1,443,708	257,368	14,228	853,477	27.01
*Salvia dugesii*	1,475,191	641,769	182,077	8,698	402,528	27.29
*Salvia elegans*	5,762,916	2,668,946	393,790	26,297	1,966,249	34.12
*Salvia farinácea*	4,399,124	1,997,356	384,195	18,243	1,523,587	34.63
*Salvia filipes*	5,580,520	2,608,588	334,714	26,475	2,122,980	38.04
*Salvia fulgens*	3,937,194	1,830,795	255,099	18,437	1,456,024	36.98
*Salvia gesneriiflora*	3,283,816	1,539,337	183,426	19,454	1,304,375	39.72
*Salvia greggii*	4,790,649	2,224,569	316,459	22,633	1,754,437	36.62
*Salvia helianthemifolia*	5,422,154	2,532,139	327,927	26,099	2,300,789	42.43
*Salvia hispanica* [10,685]	3,630,464	1,757,205	76,245	38,871	1,210,285	33.34
*Salvia hispanica* [16]	866,851	373,743	111,831	7,281	258,323	29.80
*Salvia inconspicua*	4,180,128	1,934,091	277,441	33,209	1,796,346	42.97
*Salvia involucrata*	3,456,571	1,590,456	257,524	16,111	1,201,438	34.76
*Salvia iodantha*	4,074,414	1,892,701	266,532	20,305	1,743,805	42.80
*Salvia karwinskii*	1,894,444	840,996	199,183	12,635	495,679	26.16
*Salvia keerlii*	4,646,169	2,111,991	396,028	23,818	1,656,324	35.65
*Salvia lasiantha*	3,372,169	1,561,766	215,150	31,579	1,350,572	40.05
*Salvia lavanduloides*	3,198,116	1,458,535	262,745	16,701	978,455	30.59
*Salvia leucantha*	6,810,955	3,144,365	489,051	30,542	**2,868,385**	42.11
*Salvia longispicata*	2,133,006	960,688	195,062	15,867	550,342	25.80
*Salvia longistyla*	5,501,252	2,548,398	372,042	27,520	2,141,218	38.92
*Salvia macrophylla*	4,280,359	1,972,302	312,911	17,941	1,766,499	41.27
*Salvia madrensis*	3,028,391	1,353,831	306,003	13,126	954,658	31.52
*Salvia melissodora*	4,220,056	1,950,703	282,399	33,997	1,636,602	38.78
*Salvia mexicana*	4,828,203	2,230,598	341,298	23,015	1,985,539	41.12
*Salvia microphylla*	5,107,818	2,353,701	372,947	24,491	1,929,890	37.78
*Salvia nepetoides*	3,227,624	1,474,283	242,157	35,970	983,117	30.46
*Salvia nervata*	3,113,755	1,413,887	264,897	18,405	1,087,402	34.92
*Salvia occidua*	1,722,967	773,637	155,732	19,713	453,608	26.33
*Salvia officinalis*	3,898,073	1,787,315	293,750	19,578	1,582,911	40.61
*Salvia patens*	2,250,418	1,040,462	155,325	11,942	627,819	27.90
*Salvia pauciserrata*	4,359,118	2,003,658	327,793	20,915	1,831,230	42.01
*Salvia perblanda*	2,689,851	1,190,487	293,490	13,787	1,032,314	38.38
*Salvia plurispicata*	4,256,275	1,967,417	297,047	22,526	1,789,752	42.05
*Salvia polystachia* [163]	3,825,101	1,773,511	254,027	22,340	1,495,285	39.09
*Salvia polystachia* [065]	6,430,975	2,967,587	461,330	31,384	2,757,881	42.88
*Salvia puberula*	2,241,725	979,953	267,594	13,313	562,436	25.09
*Salvia purépecha*	3,088,154	1,389,198	283,125	25,687	898,212	29.09
*Salvia purpurea* [103]	4,165,410	1,903,329	334,825	21,969	1,547,632	37.15
*Salvia purpurea* [156]	4,619,949	2,074,882	309,978	158,190	1,919,193	41.54
*Salvia ramosa*	6,793,863	3,096,212	545,286	49,591	2,624,801	38.63
*Salvia regla*	2,864,486	1,298,276	254,203	11,585	904,405	31.57
*Salvia rhyacophila*	1,073,159	512,149	30,083	18,428	282,069	26.28
*Salvia roscida*	1,818,209	771,331	261,587	13,090	405,068	22.28
*Salvia scutellarioides*	4,329,068	1,999,664	304,019	21,116	1,583,847	36.59
*Salvia semiatrata*	4,747,390	2,182,186	342,749	38,185	1,681,244	35.41
*Salvia sessilifolia*	5,376,946	2,465,483	408,983	26,462	2,137,458	39.75
*Salvia sonomensis*	3,102,641	1,433,203	218,542	14,254	1,224,534	39.47
*Salvia splendens*	6,315,864	2 945 936	386,705	31,866	2,095,385	33.18
*Salvia squalens*	6,709,903	3,086,040	499,774	28,806	2,673,573	39.85
*Salvia texana*	2,373,414	1,095,103	166,950	14,986	355,513	14.98
*Salvia tiliifolia* [5]	2,533,486	1,226,296	44,353	35,889	879,475	34.71
*Salvia tiliifolia* [15]	4,806,266	2,226,329	321,326	30,078	1,673,090	34.81
*Salvia tonaticensis*	5,311,730	2,473,768	333,255	28,388	2,018,740	38.01
*Salvia tubiflora*	6,348,744	2,916,775	480,536	28,358	2,464,460	38.82
*Salvia univerticillata*	3,756,401	1,717,023	300,936	19,717	1,033,316	27.51
*Salvia urica*	5,125,397	2,354,572	371,226	43,179	1,878,912	36.66
*Salvia vitifolia*	3,131,504	1,452,729	205,439	18,261	903,035	28.84
*Salvia wagneriana*	5,357,533	2,609,669	91,101	45,676	1,805,424	33.70
Total	337,889,127	157,329,258	20,636,288	2,393,220	1,328,625	3,085
Average	**3,754,324**	1,748,103	229,292	26,591	**1,314,368**	34.28

*Bold font indicate highest and lowest Nr. mapped reads*.

The final selection of loci for bait design was based on the following criteria: sequence variability, align-ability, demonstrated phylogenetic utility within Lamiales and Lamiaceae (Godden, unpublished data), and economic considerations. The latter criterion dictated the numbers of loci and baits per project that could be accommodated in the final multipurpose bait set. Overall, baits in the multipurpose set relevant to this project included the following: 883 Lamiales general-purpose baits (76,272 bp) and 1,207 *Salvia*-specific baits (131,394 bp), based on the Lamiales transcriptomes and *S. officinalis* and *S. splendens* alignments for the latter (Supplementary Table 2). Paired baits were manufactured with TruSeq technology by myBaits (Daicel Arbor Biosciences, Ann Arbor, MI, USA). Samples were sequenced on an Illumina® HiSeq 2,500 as 150 bp PE reads. Raw read quality was assessed with Fastqc v.0.11.2 (Andrews, 2010; Babraham Bioinformatics, Cambridge, England). Adapter sequences and low-quality bases were trimmed using Cutadapt v. 1.8.1 (Martin, 2011).

### Assembly

Raw reads were processed in HybPhyloMaker (HPM) v.1.6.4 (Fér and Schmickl, 2018), this pipeline contains multiple steps or scripts that allow assembly and further analyses (from here on throughout the text, these are quoted per acronym and numbered as specified in the script name from the HPM reference manual). Using the script HPM_0b in the pipeline, individual reads were mapped to two pseudo reference sequences. The first nuclear pseudo reference was the alignment of the probe set containing 527 putative exons (these were previously used as probes to target the specified genes) and the second pseudo reference was 114 chloroplast loci from *Salvia miltiorrhiza* Bunge complete plastome JX312195 (Qian et al., 2013), separated by 400 Ns to capture any chloroplast sequences.

**Figure 1:**
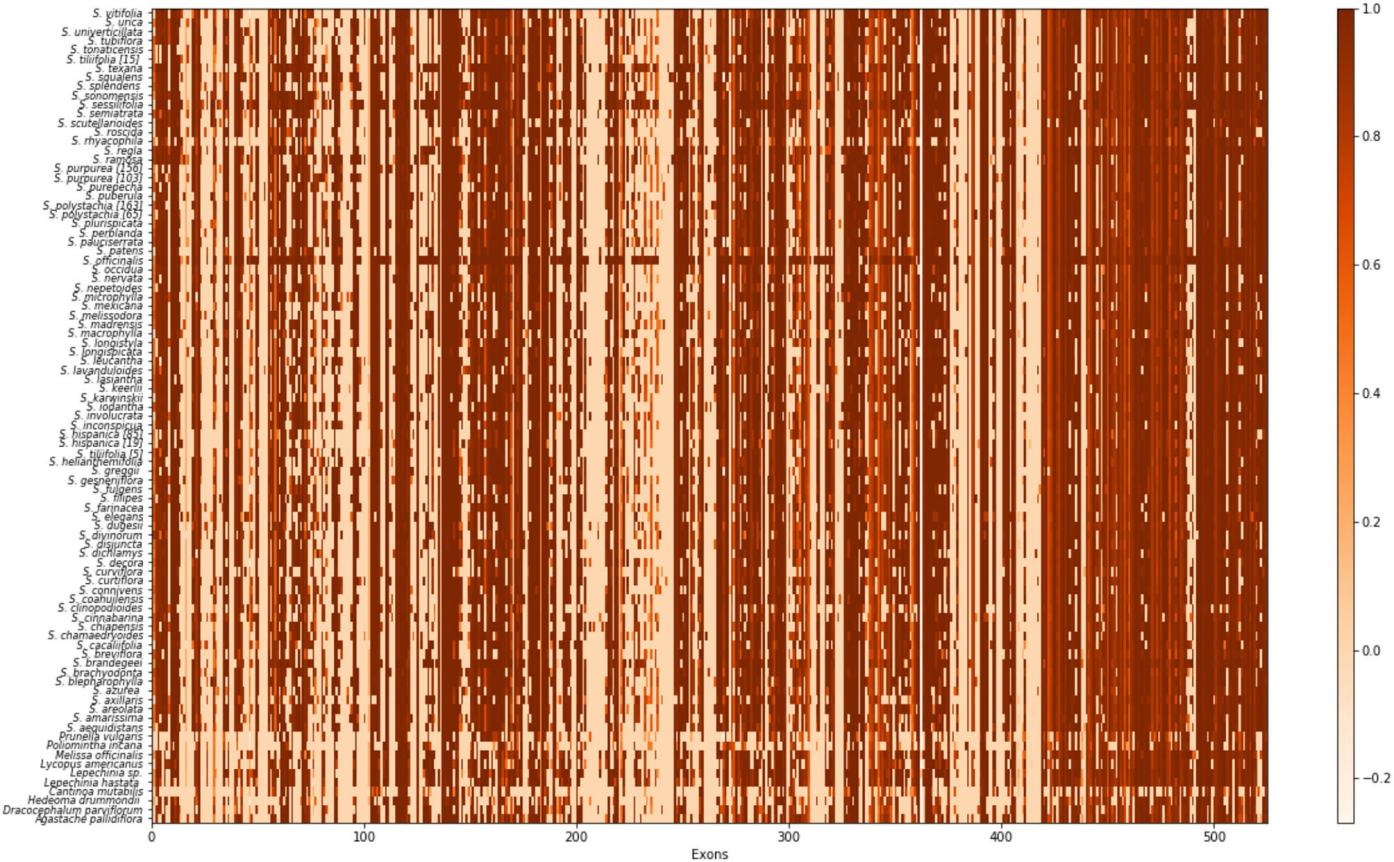
Exon recovery heat map for 527 putative exons targeted by our baits. Each column represents an exon, and each row is each species. Each cell represents the sequence completeness; a lighter color signifies fewer bases recovered for that exon and a darker color signifies more bases were recovered.

In order to summarize the effectiveness of capture based on our nuclear pseudo reference, we used all sequences for each exon produced by HPM_3 and calculated the missing data for each of them compared with the original probes in a heat map (Figure 1).

The reads were trimmed, filtered, and mapped to create the alignments for reconstructing gene and species trees, using the following steps: script HPM_1 was used to remove sequencing adapters and trim reads based on their quality using Trimmomatic v.0.33 (Bolger et al., 2014). All reads <Q20 were discarded, and the remaining reads were trimmed if the average quality in a 5 bp window was <Q20. Reads shorter than 36 bp were removed. In addition, HPM uses FastUniq v.1.1 (Xu et al., 2012) to remove duplicate reads. The script HPM_2 was used to map the quality filtered and trimmed reads to the baits pseudo reference using BWA v.0.7.16a (Li and Durbin, 2009). Mapped reads for each taxon were summarized with a consensus sequence using Kindel v.0.1.4 (Constantinides and Robertson, 2017) included in the HP pipeline. This used a 51% majority consensus rule to call bases and convert any base with low coverage (2x) to an uninformative base (N). This was repeated to consecutively map the filtered reads to the chloroplast pseudo reference.

Consensus sequences were matched to sequences of target exons using BLAT v.35 (Kent, 2002) (https://www.ncbi.nlm.nih.gov/pubmed/11932250), with 90% similarity for all samples to produce PSLX files using the script HPM_3. The script “assembled_exons_to_fastas.py” (Weitemier et al., 2014) is used in the script HPM_4a to construct matrices for multiple alignments and add Ns for taxa that lack a particular exon. Also, with the script HPM_4a, sequences were aligned in MAFFT v. 7.305 (Katoh and Standley, 2013) and nuclear exons belonging to the same gene were concatenated using AMAS (Borowiec, 2016). Finally using the script HPM_5 taxa, we took a conservative approach and removed exons from the alignment if more than 70% of the sequence missing and if exons were recovered in fewer than 75% of the taxa. We also tested the effect of this approach by varying our criterion to 30, 50, and 75% missing data for loci shared by all species in the HPM_5 matrix.

The two resulting data sets comprised 119 targeted nuclear genes and 114 loci for the chloroplast. Both data sets were independently filtered as described above to remove genes from the alignment with excessive missing data. After filtering, the alignments included 96 nuclear genes and 114 chloroplast loci.

### Phylogenetic Analyses

Bayesian and Maximum Likelihood (ML) multispecies coalescent-based approaches were used to reconstruct species trees for the nuclear data. For Bayesian inference, we used BEAST v. 2.5.2 (Bouckaert et al., 2019) for the genes obtained from the HPM pipeline. First, the best fitting molecular evolution model was obtained for each independent gene using jModelTest v. 2.1.10 (Darriba et al., 2012). Four models were selected as best fitting (GTR + G, HK + G, K80 + G, and SYM + G). We ran BEAUTI v. 2.5.2 (Bouckaert et al., 2014) using the template for StarBEAST to prepare the BEAST analysis input file. In the analysis, trees were unlinked and the strict clock model was used for all of them. Genes with the same molecular evolution model had linked parameters. Finally, a coalescent constant population model was used as a *prior* on the species tree. We ran BEAST for 1.6 B states, sampling every 5,000 states. Tracer v. 1.6 (Drummond and Rambaut, 2007) was used to check ESS values. To construct a maximum clade credibility tree, we used TreeAnnotator v. 2.5.6 (Bouckaert et al., 2019) setting a burn-in of 25% of the states and “Mean Height” for node heights.

For ML inference, we used the scripts HPM_6b and HPM_7, that execute FastTree 2.1.10 SSE3 (Price et al., 2010) using default parameters, to generate trees for every gene in our dataset and root them using the external group (*Cantinoa*). Next, the species tree was inferred using the coalescent-based approach implemented in ASTRAL-III v. 5.6.1 (Zhang et al., 2018) running the script HPM_8a with default parameters. To reconstruct the phylogenetic network, we used the 96 gene trees produced by HPM_7 as input to NANUQ (Allman et al., 2019) incorporated in the MSCquartets package (Rhodes et al., 2021) for R (R Core Team, 2017, Vienna, Austria). We set an alpha of 1e-5 and a beta of 0.95 with the goal of testing for a signal of network cycles in the quartets. Later, we used SplitsTree (Huson and Bryant, 2006) to plot the network using default parameters.

To test the robustness of the phylogenetic inferences obtained for both nDNA and cpDNA matrices, we compared trees with different percentages of missing data (30, 50, and 75% missing), and a tree that maintains loci for all the samples (as opposed to removing loci present in fewer than 75% of taxa). For each dataset with different missing data, we re-ran the nuclear ASTRAL reconstruction and the chloroplast FastTree analysis with the parameters described earlier.

## Results

### Bait Success and Assembly

After removing low-quality sequences and loci with many missing taxa in HPM, 96 of 119 genes targeted by our respective bait sets were retained for analysis. Samples had an average of 1,314,368 mapped reads (Table 1), with the fewest in *S. breviflora* Moc. and Sesse ex Benth. (255,228 reads) and the most in *S. leucantha* Cav. (2,868,385 reads). The length of nuclear gene alignments ranged from 154 bp (AT1G05350) to 3,336 bp (AT4G19490). In total, 527 putative exons were recovered. However, about a third of the targeted exons were retained for further analysis (Supplementary Tables 3, 4). The filtering step in HPM removed some of the 527 putative exons, given that exon capture was not homogeneous across all samples nor loci. Fewer base pairs were recovered for the outgroup than the in-group and the highest recovery was in *S. officinalis*, one of the transcriptomes used to design the *Salvia* baits.

The HPM chloroplast assembly for all 90 samples, using the *S. miltiorrhiza* genome (Qian et al., 2013) as a pseudoreference, recovered 75 CDS (59 in the LSC, 5 IR-B, 10 SSC, 1 IR-A), 29 tRNA (20 LSC, 7 IR-B, 1 SSC), 5 genes with introns (3 LSC, 1 IR-B, 1 SSC), 4 rRNA in the IR-B and 1 IGS in the LSC region (Supplementary Table 5); ranging in length from 36 bp (*rps*19) to 6,870 bp (*ycf* 2).

### Phylogenetic Inferences

**Figure 2:**
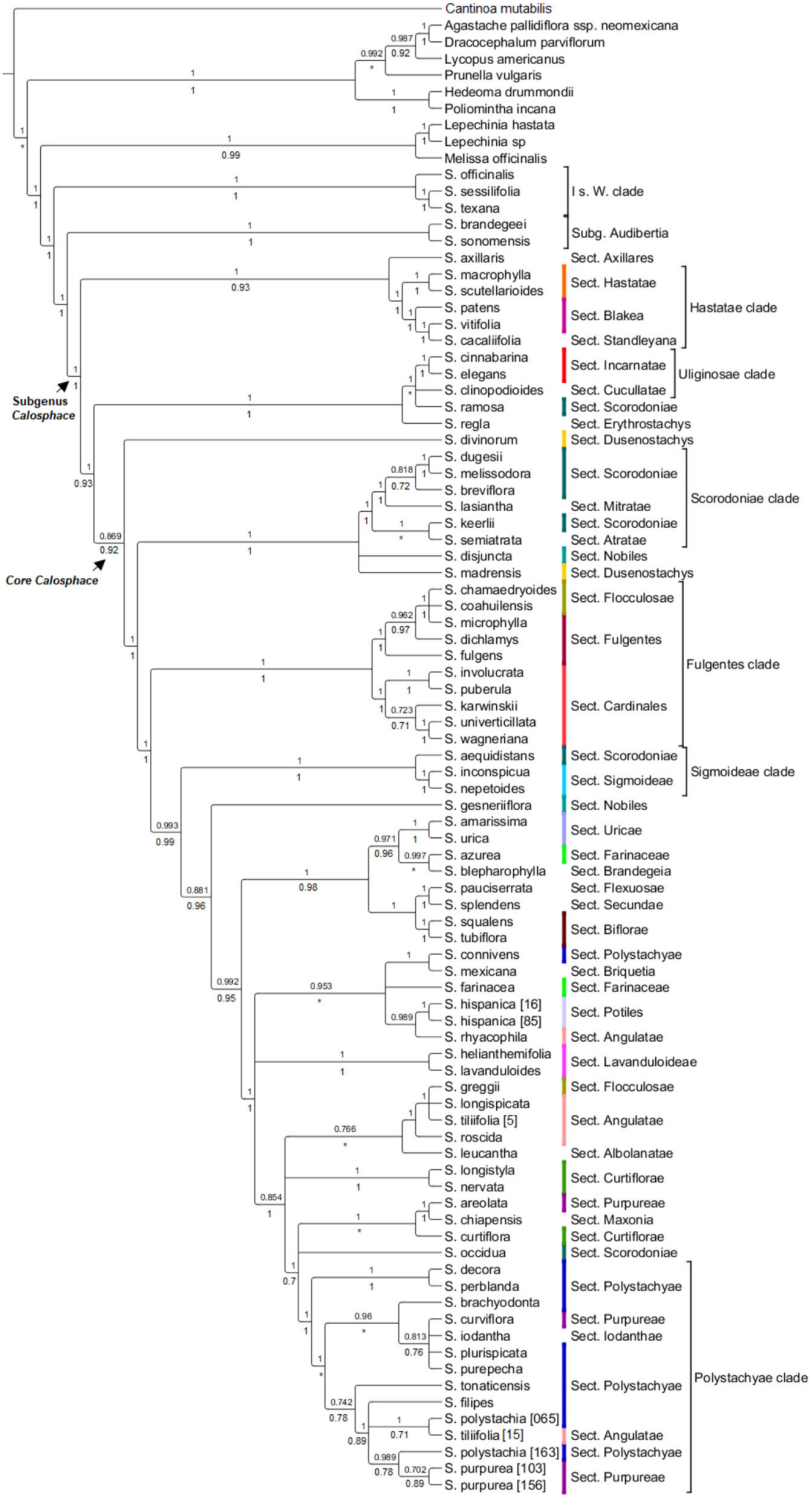
HPM- BEAST tree for 96 nuclear genes with up to 70% missing data allowed for each exon. The BEAST local posterior probability is indicated above branches from the analysis and the ASTRAL analysis is under the branches. Branches with support values <0.7 are collapsed. *Salvia* subgenus *Calosphace* sections *s*. Epling are color-coded. The main clades follow previous nomenclature (Walker et al., 2004; Jenks et al., 2013; Fragoso-Martínez et al., 2018).

**Figure 3:**
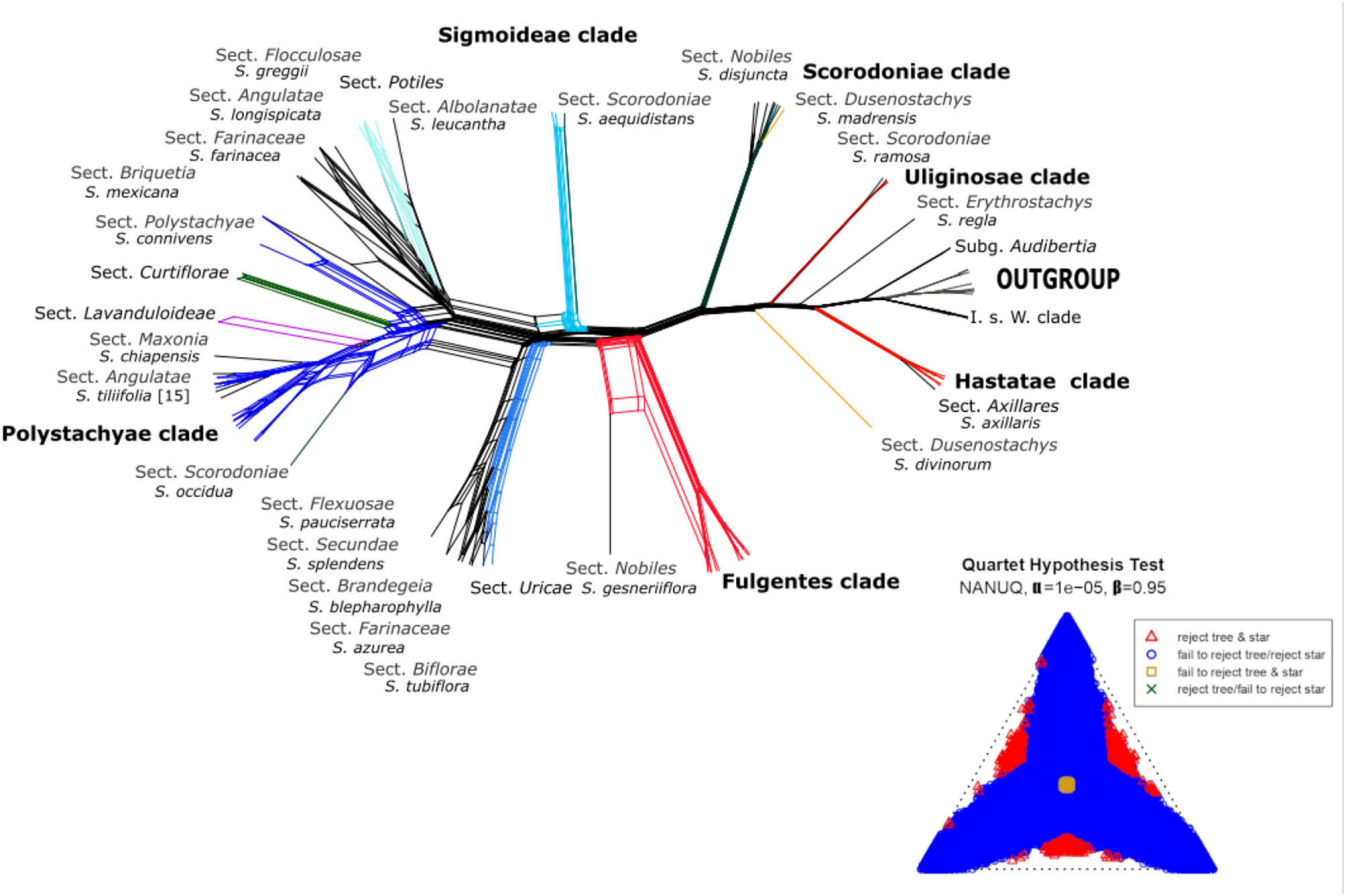
Network analysis for 527 putative nuclear exons with *S*. subg. *Calosphace* sections are color-coded as in Figure 1. A simplex plot of NANUQ's quartet hypothesis test at α = 0.0001 and ß = 0.95.

All nuclear phylogenetic inferences, with both coalescent analyses HPM [BEAST (Figure 2) and ASTRAL (Supplementary Figure 1)] recovered similar tree topologies, with some differences in shallow-level relationships. A network of the nuclear alignment (Figure 3) revealed the same groupings in the outgroup and some reticulation within the core *Calosphace* as we recovered in our phylogenetic analyses. A quartet hypothesis test showed that a majority of quartets had a tree-like signal, with only a few quartets better represented as four-cycle networks (Figure 3). We also tested if varying the missing data to 30 (Supplementary Figure 2A), 50 (Supplementary Figure 2B), or 70% (Supplementary Figure 2C) would have an impact on the overall tree topologies (Supplementary Figure 1), but there were no major differences in the topologies and only differences in support values for some branches. Species relationships in the broader Lamiaceae HPM assembly were rooted with *C. mutabilis* (tribe Ocimeae), followed by a clade which includes *Dracocephalum, Agastache, Lycopus*, and *Prunella* (1 local posterior probability [localPP] in every three), a second sister clade with *Poliomintha* and *Hedeoma* (1 localPP), and the third clade with *Melissa* and *Lepechinia* (Figure 2). The four *Salvia* subgenera sampled (Figures 2, 3; Supplementary Figures 1 and 2) are in “clade I” (clade nomenclature *sensu*; Walker et al., 2004; Jenks et al., 2013) with 1 localPP in every inference. Clade 1 included *S*. subg. *Salvia* (*S. officinalis*) and *Leonia* (*S. sessilifolia* and *S. texana*), sister to a clade of *S*. subg. *Audibertia* and *Calosphace* (1 localPP).

There were 8 out of the 13 *Salvia* subg. *Calosphace* sections *sensu* Epling which were sampled here and represented by more than one sample were monophyletic in all analyses (Table 2). They include *Cardinalis, Biflorae, Hastatae, Incarnatae, Lavanduloideae, Sigmoideae*, and *Uricae*, while *Curtiflorae* was only monophyletic in the nuclear ASTRAL and FastTree trees.

**Table 2:** *Salvia* subgenus *Calosphace* monophyletic sections *s*. Epling, comparative of previous phylogenetic analysis and our Hyb-seq three nuclear and chloroplast analyses.

***Salvia* sect. *sensu* Epling**	**Fragoso-Martínez et al. (2018)**	**nASTRAL**	**nBEAST**	**cpFastTree**
*Angulatae* (4/52)	No	No	No	No
*Biflorae* (2/4)	Yes	Yes	Yes	No
*Blakea* (2/5)	Yes	No	No	No
*Cardinalis* (5/9)	No	Yes	Yes	Yes
*Curtiflorae* (3/9)	Yes	Yes	No	No
*Fulgentes* (3/9)	No	No	No	No
*Hastatae* (2/7)	Yes	Yes	Yes	Yes
*Incarnatae* (2/2)	Yes	Yes	Yes	Yes
*Lavanduloideae* (2/18)	Yes	Yes	Yes	Yes
*Polystachyae* (9/16)	No	No	No	No
*Purpureae* (3/9)	No	No	No	No
*Sigmoideae* (2/9)	Yes	Yes	Yes	Yes
*Uricae* (2/2)	No	Yes	Yes	Yes

Several clades within *S*. subg. *Calosphace* was well-resolved and strongly supported by our phylogenetic results. A “*Hastatae* clade” with 1 PP (ASTRAL/BEAST) includes members of the *S*. sects. *Hastatae, Blakea*, and *Standleyana* are sisters to *S. axillaris* (monotypic *S*. sect. *Axillares*) (Figure 2). The “*Uliginosae* clade” includes a monophyletic *S*. sect. *Incarnatae* (*Salvia elegans* Vahl. + *Salvia cinnabarina* M. Martens and Galeotti) in all the analyses (1 localPP), and one sample each in *S*. sects. *Erythrostachys* (*Salvia regla* Cav.), *Cucullatae* (*Salvia clinopodioides* Kunth) and *Scorodoniae* (*Salvia ramosa* Brandegee). Following these *Calosphace* clades, we reached the “core *Calosphace”* (64 of the remaining species), where resolution and clade support are variable in the nuclear phylogenetic inferences (Figures 2, 3; Supplementary Figures 1, 2).

Within the “core *Calosphace,”* the highly supported clades (1 localPP) included the “*Scorodoniae* clade” with species in *S*. sects. *Atratae, Mitratae*, and *Scorodoniae*. A large “*Fulgentes* clade” with monophyletic *S*. section *Cardinales* (with five of its nine species sampled) and some members of *S*. sects. *Fulgentes* and *Flocculosae* (1 local PP BEAST/ASTRAL). The “*Sigmoideae* clade” (1 local PP BEAST/ASTRAL) with *Salvia inconspicua* Benth. + *Salvia nepetoides* Kunth. and *Salvia aequidistans* Fernald (*S*. sect. *Scorodoniae*); a large clade with *Salvia gesneriiflora* Lindl. and Paxton in *S*. sect. *Nobiles* from Walker's “*Fulgentes* clade” [BEAST (0.881 local PP); ASTRAL (0.96 local PP)] and smaller strongly supported clades (1 local PP BEAST/ASTRAL) including monophyletic *S*. sects. *Uricae*, “*Lavanduloideae* clade,” and “*Biflorae* clade,” while *Curtiflorae* only in ASTRAL (0.96 localPP). Finally, the “*Polystachyae* clade” (1 localPP BEAST/ASTRAL), includes representatives from the *S*. sect. *Angulatae* (*S. tiliifolia*), *Iodanthae* (*Salvia iodantha* Fernald), *Polystachyae* (*S. brachyodonta, S. decora, Salvia filipes* Benth., *S. perblanda, Salvia plurispicata* Epling, *S. polystachia, S. purepecha, Salvia tonaticensis* Ramamoorthy ex Lara-Cabrera, Bedolla and Zamudio), and sect. *Purpureae* (*Salvia curviflora* Benth. and *S. purpurea*) and two samples each for *S. polystachia* (non-monophyletic) and *S. purpurea*, (monophyletic; Figures 2, 3; Supplementary Figure 1).

The ML concatenated FastTree of the chloroplast loci (Figure 3) for the 90 samples, provided high support (1 localPP) for deep-level relationships within the *Ocimiae* and *Menthae*, and a sister relationship between *S*. subgenera *Audibertia* and *Calosphace*. Well-resolved and highly supported clades in this tree include *S. axillaris* as sister to the rest of subg. *Calosphace*; the “*Hastatae* clade” (1 localPP) and “*Uliginosae* clade” (1 localPP), with monophyletic *S*. sect. *Cardinales* (0.99 localPP), *Hastatae* (1 local PP), *Incarnatae* (1 local PP), and *Uricae* (1 localPP), and *S. hispanica* (two sampled). However, resolution and clade support are reduced for a few of the “core *Calosphace*,” such as the *S. genesneriflora* polytomy and *S. sect*. *Cucullatae* + *Scorodoniae, Flexulosae, Farinaceae, Albolanatae*. Two sections are not monophyletic for the cpDNA data *Lavanduloides* and *Sigmoideae*.

## Discussion

### NGS in *Salvia*

The Hyb-Seq protocol (Weitemier et al., 2014) implemented, here, resolved deep phylogenetic relationships in Lamiaceae, among *Salvia* subgenera, and within a recently diverged *S*. subg. *Calosphace* (Figure 2; Supplementary Figure 1), providing additional support for existing phylogenetic hypotheses (Walker, 2006; Jenks et al., 2013; Fragoso-Martínez et al., 2018). We enriched 119 nuclear loci (Supplementary Tables 3, 4), 96 of which were left for phylogenetic estimations after the HPM filtering process.

To date, this is the largest base-pair sampling for this many *Salvia* species using the next-gen technology and specifically designed baits, and we were able to recover 1,314,368 bp (Table 1) in the HPM assembly for 96 nuclear genes in all 90 Lamiaceae sampled (14,604 b per sample; Supplementary Table 4). Previous anchored hybrid enrichment experiments in *Salvia* sampled 12 species for 453 loci producing a final alignment of 282,219 bp or 23,518 bp per sample (Fragoso-Martínez et al., 2017). Another study sampled 35 species (13 *Calosphace*) for 438 loci with a final alignment of 272,874 bp or 7,796 bp per sample (Kriebel et al., 2019). The studies by Fragoso-Martínez et al. (2017) and Kriebel et al. (2019) reported higher numbers of loci and base pairs than we did, but with less than half of our sampled taxa. Our methods had a more stringent cut-off for missing sequences and yielded a more conservative alignment. The branches in our tree with low support led to taxa that were not sampled in the study of Fragoso-Martinez or Kriebel et al. (2019).

We did not attempt a direct comparison between our custom-designed baits and previous next-gen studies using bait selection in Angiosperm v.1 kit (Buddenhagen et al., 2016). These three studies had different taxon sampling and phylogeny estimation methods so, it is not clear if the differences we report on branch support derive from our baits or taxon sampling.

### Chloroplast Assembly

An additional advantage of the Hyb-Seq protocol as opposed to the AHE protocol, lies in obtaining the chloroplast and mitochondrial genomes, here we explored the chloroplast loci. Chloroplasts were assembled in HPM using *S. miltiorrhiza* genome as a pseudoreference, obtaining a 92,461 bp assembly for the 90 *Salvia* samples evaluated (Supplementary Table 5). A map to reference approach was previously tested (Olvera-Mendoza et al., 2020) on 15 samples from these same data to investigate closely related species in *S*. sections *Atratae, Mitratae, Scorodoniae*, and *Sigmoideae*, resulting in the first chloroplast genome assemblies for *S*. subg. *Calosphace*, although limited taxon sampling for these sections impeded full resolution of the phylogeny. Our HPM chloroplast assembly using the same pseudoreference recovered fewer loci (Supplementary Table 5) than the study conducted by Olvera-Mendoza et al. (2020) did [114 genes, 80 CDS, 30 tRNA spacers, and 4rRNA's (Olvera-Mendoza et al., 2020) vs. our 75 CDS, 29 tRNA's, 5 introns and 4 rRNA]. This may be attributed to the many samples (78) we evaluated compared with their 15 samples, and the filtering step we used during HPM.

### Nuclear Phylogenetic Inferences

The nuclear phylogenies (Figure 2; Supplementary Figure 1) resulting from ASTRAL and BEAST have well-resolved and highly supported clades and recover several previously reported relationships (Walker et al., 2004; Walker and Sytsma, 2007; Jenks et al., 2013; Will and Claßen-Bockhoff, 2017; Fragoso-Martínez et al., 2018). *Cantinoa* from tribe *Ocimeae* was used as the outgroup following Li et al. (2016). *Cantinoa* is sister to the *Mentheae* tribe and relationships in our trees are in agreement with the study of Drew and Sytsma (2012). We recovered subtribes Menthinae (*Hedeoma* and *Poliomintha*), Nepetinae (*Agastache* and *Dracocephalum*), Lycopinae (*Lycopus*), Prunellinae (*Prunella*), and Salviinae (*Melissa*, both *Lepechinia* and *Salvia*). Within *Salvia*, we recovered “clade I” with *S*. subgenera *Salvia* (*S. officinalis*) and *Leonia* (*S. sessilifolia* + *S. texana*), and a clade of *S*. subgenera *Audibertia* (*S. sonomensis* + *S. brandegeei*) and the 69 remaining species in *Calosphace*. Here we support the monophyly of eight of the 13 *Salvia* sections sampled (Table 2): *Biflorae, Curtiflorae, Hastatae, Incarnatae, Lavanduloideae, Sigmoideae*, and also *S*. sections *Cardinales* and *Uricae* (as in Olvera-Mendoza et al., 2020). Although our tree is well-resolved, our *Calosphace* sample is <15% of the estimated species diversity in the subgenus, undoubtedly having an effect on clade resolution, and unsampled species could potentially be inserted in future phylogenetic studies to further resolve fine-scale relationships with each clade.

**Figure 4:**
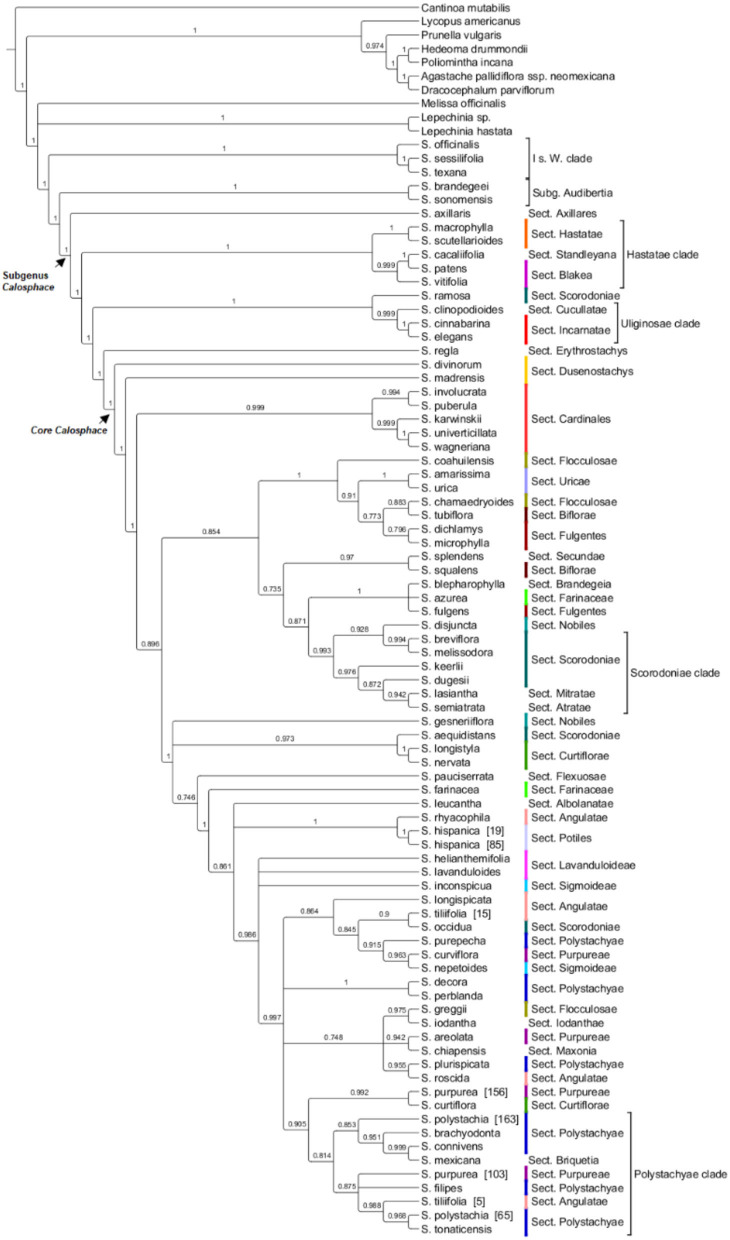
HPM- ML FastTree for 114 chloroplast loci, local posterior probability >0.7 is indicated above branches (lower are collapsed). *Salvia* subgenus *Calosphace* sections *s*. Epling are color-coded. The main clades follow previous nomenclature (Walker et al., 2004; Jenks et al., 2013; Fragoso-Martínez et al., 2018).

Relationships among the section's sister to the core *Calosphace* have been somewhat controversial. Most studies (Walker et al., 2004; Walker and Sytsma, 2007; Jenks et al., 2013; Fragoso-Martínez et al., 2018; Kriebel et al., 2019) found *S. axillaris* (monotypic *S*. sect. *Axillares*) sister to the rest of the *Calosphace*; this relationship is only supported by our chloroplast analysis (Figure 4). Our nuclear data analyses (Figure 2; Supplementary Figure 1) support *S. axillaris* sister to “*Hastatae* clade,” and together with sister to the rest of *Calosphace*; this relationship has also been recovered by Hu et al. (2018) [(*S. patens* + *Salvia cacaliifolia* Benth. (*S*. *axillaris* (rest of *Calosphace*)] and Walker et al. (2015) [(*S. patens* (*S. axillaris* + *Salvia cedrosensis* Greene)] with *S. axillaris* in a clade with *Hastatae* representatives. Interestingly, these relationships are congruent with differences in stamen morphology; a key feature in *Salvia* (Bentham, 1832-1836; Fernald, 1900; Walker and Sytsma, 2007). Three stamen types have been described for *S*. subg. *Calosphace*; the G type in *S. axillaris* where both anterior and posterior anthers are expressed in free stamens, F type in the “clade Hastatae” (*S*. sects. *Standleyana, Blakea*, and *Hastatae*) where “both posterior thecae are aborted, and the adjacent posterior thecae are not, or only little fused” (Walker and Sytsma, 2007) and the E stamen type in the rest of the *Calosphace* where the posterior anthers are aborted and stamens are joined in a connective (Walker and Sytsma, 2007). The relationship we recovered suggests that elaborated connective tissue may have evolved twice in this clade (in *Hastatae* and *Calosphace*) or that the ancestor of the clade had another connective and it was lost in *S. axillaris*. The complex evolutionary patterns of stamen morphology are being investigated (Kriebel et al., 2020), to consider the potential usefulness of stamen characters for defining clades and within-species variation.

Previous next-gen studies of *Salvia* by Fragoso-Martínez et al. (2017) used the angiosperm bait kit (Johnson et al., 2019) and found three branches with low posterior probability (PP) support (their Figure 1b). Kriebel et al. (2019) on the other hand, found three poorly supported branches in the *Calosphace* clade in their ASTRAL coalescent analysis (Figure 2). We did not sample the taxa involved in two of those branches. Kriebel et al. (2019) additionally report an expanded taxon sampling to 266 *Calosphace* merging previous nuclear ribosomal DNA (ITS/ETS) sequences as supporting material for their habitat and pollinator study for *Salvia*.

Clade “*Hastatae*” was recovered in every tree (Figures 2, 4; Supplementary Figures 1–3) and includes reciprocally monophyletic *S*. sects. *Hastatae, Blakeae*, and *Standleyana*. This clade was also found in other studies [Jenks et al., 2013; Fragoso-Martínez et al., 2018; Kriebel et al., 2019 (nrDNA)]. *Salvia* sect. *Standleyana* was redefined by Turner (2011), merging it with species from the *S*. sect. *Blakea s*. Epling (*Salvia costaricensis* Oerst., *S. patens, S. subpatens* Epling, *S. vitifolia*). Later Klitgaard (2012) supported the merger of these *S*. sections, but under sect. *Blakea*. Our phylogenies found *S. cacalifolia* in a clade with *S. patens* and *S. vitifolia* and so support the merger of sect. *Standleyana* and *Blakea*, with the caveat, that *S. costaricensis* Oerst., *S. subpatens*, and *S. serboana* B. L. Turner should be sampled in a molecular study before the sections are re-classified.

Our clade “*Uliginosae*” (Figures 2, 4; Supplementary Figures 1–3) includes monophyletic *S*. sects. *Incarnatae* and *Cucullatae*, agreeing with previous clade circumscription [Jenks et al., 2013; Fragoso-Martínez et al., 2018; Kriebel et al., 2019 (nrDNA)]; unfortunately, though, our sampling in this clade is reduced, and we are lacking a representative of *S*. sect. *Uliginosae*; furthermore, our trees include *S. regla* and one of the seven sampled *S*. sect. *Scorodoniae* (*S. ramosa*) in the *S*. sect. *Erythrostachys* clade; these relationships require careful review with broader taxon sampling within the *S*. sect. *Erytrostachys*.

Following clades “*Hastate*” and “*Uliginosae*” we reach the troublesome and most species-rich clade, the “core *Calosphace”* (Figures 2–4; Supplementary Figures 1–3). The remainder of the sampled species is included in this clade. Walker (2006) was the first to define this clade, consisting of several clades immersed within a large polytomy and later studies with expanded sampling have confirmed this clade (Jenks et al., 2013; Fragoso-Martínez et al., 2018). We newly placed seven species in the *Calosphace* clade, classified in the *S*. sects. *Angulatae* (*S. roscida*), *Cardinales* (*S. puberula*), *Fulgentes* (*S. dichlamys*) and *Polystachyae* (*S. brachyodonta, S. decora, S. perblanda, S. purepecha*). Additionally, we found monophyletic *S*. sects. *Cardinalis* and *Uricae*, increasing the molecular evidence for monophyletic *Calosphace* sections from 12 (Fragoso-Martínez et al., 2018) to 14 among those evaluated.

A close sectional relationship has been demonstrated for *Salvia* sects. *Scorodoniae Atratae* (*S. semiatrata*), *Mitratae* (*Salvia lasiantha* Benth.), *Sigmoideae* (*S. inconspicua* and *S. nepetoides*), and *Uricae* (*S. amarissima* and *S. urica*) cpDNA entire genome and nuclear ribosomal cistron (Olvera-Mendoza et al., 2020). We found support for relationships among some of these sections, but together they do not form a clade; *S*. sect. *Uricae* is indeed monophyletic and distinct from the *S*. sect. *Scorodoniae* as Olvera-Mendoza et al. (2020) proposed. *Salvia* sect. *Scorodoniae* is not monophyletic although morphologically recognizable (Olvera-Mendoza et al., 2017) and *S*. sect. *Sigmoideae* is monophyletic only if nuclear data are incorporated in the analysis, either combined cpDNA + nDNA (Jenks et al., 2013; Fragoso-Martínez et al., 2018; Olvera-Mendoza et al., 2020) or only nuclear (Figure 2; Supplementary Figures 1–3A–C); highlighting the importance of nuclear markers to better-resolve *Salvia* species relationships. Jenks et al. (2013) and Fragoso-Martínez et al. (2018) also recovered a non-monophyletic *S*. sect. *Scorodoniae* [as did Kriebel et al., 2019 (nrDNA)] and considered *S*. sect. *Uricae*'s species to be best placed within *S*. sect. *Scorodoniae*. It is clear that further analysis is required to solve species relationships within these sections, strive to fully sample *S*. sects *Scorodoniae* and *Sigmoideae*, coupled with a thorough morphological review.

Our topology for “*Fulgentes* clade” (Figure 2; Supplementary Figure 1) is similar to previous inferences but with high branch support (Jenks et al., 2013; Fragoso-Martínez et al., 2018; Kriebel et al., 2019 [nrDNA]) for the nuclear loci analyses, including members in *S*. sects. *Fulgentes, Flocculosae*, and *Cardinalis* (their *Holwaya s*. Ramamoorthy, 1984). Fragoso-Martínez et al. (2017) AHE analysis report *S. fulgentes* sister to the rest of core *Calosphace* except *S. melissodora* and *S. mocinoi* in their branch B3 (0.71 PP). *Salvia* sect. *Cardinales* is here represented by five (*Salvia involucrata* Cav., *Salvia karwinskii* Benth., *S. puberula, Salvia wagneriana* Pol., *Salvia univerticillata* Ramamoorthy ex Klitg.) of its nine species and is monophyletic and strongly supported in all nuclear (Figure 2; Supplementary Figures 1, 2A–C) and chloroplast trees (Figure 4; Supplementary Figures 3A–C). Section *Cardinales* is sister to a clade of *S*. sects. *Fulgentes* (*Salvia fulgens* Cav., *S. dichlamys, Salvia microphylla* Kunth) and *Flocculosae* (*Salvia chamaedryoides* Cav., *Salvia coahuilensis* Fernald), only our *Salvia greggii* A. Gray (*S*. sect. *Flocculosae*) is apart from this clade. Despite the non-monophyly of *S*. sects. *Flocculosae* and *Fulgentes* we agree with Jenks et al. (2013) on their morphological and phylogenetic relationships.

One of the most species-rich sections in *Salvia* subg. *Calosphace* is *Angulatae* (52 species) and it is also one of the most morphologically complex and has a disjunct distribution in N and S America (Epling, 1939; Walker, 2006). None of the previous studies have recovered it as monophyletic [Walker, 2006; Jenks et al., 2013; Fragoso-Martínez et al., 2018; Kriebel et al., 2019 (nrDNA)]. Here we found three species, *S. roscida, S. longispicata and S. tiliifolia* [5] form the broadly defined “*Angulatae* clade” (Figure 2; Supplementary Figure 1) and *S. tiliifolia* [15] is sister to *S. polystachia* [163] within the “*Polystachyae* clade.” The non-monophyly of *S. tiliifolia* is both troublesome and expected since Walker (2006) found a monophyletic *S. tiliifolia* lacking bootstrap support in his neighbor-joining tree, and *S. tiliifolia* is one of the most broadly distributed and morphologically complex species in subg. *Calosphace*. Section *Angulatae* is in urgent need of a thorough review, both morphologically and molecularly; to date, only 22 South American members have been studied (Fernández-Alonso, 2003; Wood, 2007) and there are ~26 North American members that remain to be sampled.

Finally, the “*Polystachyae* clade” (Figures 2–4; Supplementary Figures 1–3) includes members from *S*. sects. *Angulatae* (*S. tiliifolia* [15]), *Curtiflorae* (*S. curtiflora*), *Iodanthae* (*S. iodantha*), *Maxonia* (*Salvia chiapensis* Brandegee)*, Purpureae* (*S. curviflora, S. purpurea*), and *Scorodoniae* (*S. occidua*). Three of these sections have been under study for some time since Walker (2006) first found *S. iodantha, S. polystachia*, and *S. purpurea* in a clade with only 1-2 bp difference in *psbA-trnH, trnL-trnF*, and ITS sequences. Later Bedolla-García (2012) expanded taxon sampling and regarded this as the “PIP clade,” due to the inclusion of members of *S*. sects. *Purpureae* from Mexico (*S. areolata*, S. *curviflora, S. littae, S. purpurea, S. raveniana*), *Iodanthae* (*S. iodantha*, considering *Salvia arbuscula* Fernald and *Salvia townsendii* Fernald as synonyms) and *Polystachyae* (*S. brachyodonta, Salvia connivens* Epling, *Salvia compacta* Kuntze, *S. decora, S. filipes, Salvia mcvaughii* Bedolla, Lara Cabrera and Zamudio, *S. plurispicata, S. polystachia, Salvia tonalensis* Brandegee, *S. tonaticensis*). Here we include nine of the sixteen species in the *S*. sect. *Polystachyae*, three species of *S*. sect. *Purpureae* and *S. iodantha* (sole species in *S*. sect. *Iodanthae*), and all sampled taxa of these sections, with the exception of *S. connivens* (*S*. sect. *Polystachyae*), are in this clade. Neither *S*. sects. *Purpureae* nor *Polystachyae* are monophyletic, as has been the case elsewhere [Jenks et al., 2013; Fragoso-Martínez et al., 2018; Kriebel et al., 2019 (nrDNA)]. For this troublesome, widely diverse clade we recovered only one consistent and supported sister relationship (*S. decora* and *S. perblanda*) in the nuclear trees (Figures 2, 3; Supplementary Figures 1, 2), network (Figure 3), and also in the cpDNA tree (Figure 4; Supplementary Figure 3). Otherwise, species relationships in this part of the tree have less support, with some polytomies and low to medium branch support (Figures 2, 3; Supplementary Figures 1, 2). This lack of branch support and the network results strongly suggest reticulation issues due to recent divergence, hybridization, or incomplete lineage sorting (Huang et al., 2017). Additionally, we found that *S. purpurea* is monophyletic in the nuclear evidence, whereas *S. polystachia* is not.

Aside from the main clades “*Hastatae,” “Uliginosae*,” “*Scorodoniae*,” “*Fulgentes*,” “*Sigmoideae*,” and “*Polystachyae”* we found other strongly supported, small clades. *Salvia* sect. *Uricae* is monophyletic and *S*. sects. *Farinaceae, Nobiles*, and *Dusenostachys* are non-monophyletic, as has been previously reported [Jenks et al., 2013; Fragoso-Martínez et al., 2018; Kriebel et al., 2019 (nrDNA)]. We also support the monophyly of *Salvia hispanica* (*S*. sect. *Potiles*), the two samples forming a clade with *S. rhyacophila* (*S*. sect. *Angulatae*) as did Fragoso-Martínez et al. (2018); whereas Fragoso-Martínez et al. (2017) AHE analysis found a poorly supported sister relationship between *S. hispanica* and *S. heliamenthifolia* (0.53).

### Chloroplast Phylogeny

Following Doyle (2021) we opted to analyze our chloroplast data as a single hereditary unit through ML in FastTree (Figure 3). The chloroplast tree supports the outgroup relationships *S. axillaris* as sister to the rest of *S*. subg. *Calosphace* and sister lineages and clades “*Hastatae*” and “*Uliginosae*,” and monophyletic *S*. sects. *Cardinales, Hastatae, Incarnatae* and *Uricae*. Only two *S*. sects. are not monophyletic here as opposed to nDNA, *Lavanduloides*, and *Sigmoideae*. Our nuclear and chloroplast analyses, however, used distinct pseudo references, here, we used the distantly-related *S. miltiorrhiza* (*Salvia* subg. *Sclarea* sect. *Drymosphace* Hu et al., 2018) as the chloroplast assembly pseudoreference. *Salvia miltiorrhiza* is sister to clade *Meriandra* + *Dorystaechas* + *Ramona* (*Salvia* subg. *Audibertia*) + *Lasemia* (*Salvia* subg. *Calosphace*) (Will and Claßen-Bockhoff, 2017).

Our nuclear and chloroplast phylogenies are in overall agreement, for the outgroup, sister relationship of *Audibertia* and *Calosphace* and well-resolved “*Hastatae*,” “*Uliginosae*,” “*Scorodoniae*,” and “*Polystachyae*” clades. However, they disagree on the placement of *S. axillaris* as sister to “clade *Hastatae*” in nuclear trees or sister to the rest of the *Calosplace* in the chloroplast tree. Within the core *Calosphace*, particular complexity in the phylogenies and network is seen with *Salvia gesneriiflora*, a bird pollinated and morphologically distinct species. This species is one of the two representatives of the *S*. sect. *Nobiles* in our sampling (*S. disjuncta* is the other) and S. *gesneriiflora* placement moves between the “*Sigmoideae*” and “*Uricae* clades” in BEAST (Figure 2), between the “*Fulgentes* clade” and “*Sigmoideae* clade” in ASTRAL (Supplementary Figure 1), and between the “*Scorodoniae* clade” and *Scorodoniae*+*Curtiflorae* clade in the chloroplast tree (Figure 4). Furthermore, the network shows the nuclear loci for this species have characters that align it with *S. coahuilensis* in clade *Flocculosae* + *Uricae* + *Fulgentes* and also align it with the remaining core *Calosphace* clade (Figure 3). It is unclear why the placement of this particular species is so troublesome, no hybridization events have been reported, though frequent nectar robbing does occur (Cuevas et al., 2013), so hybridization may be a possibility worth further exploration. It is possible that we lacked sampling of phylogenetically closer relatives. Interestingly, the sectional circumscription of this species has also been controversial, Santos (1991) moved *S. gesneriiflora* from the *S*. sect. *Nobiles* Epling (1939) to sect. *Holwayana*. Testing the placement of this species would require a phylogeographic approach.

### Species Monophyly

This research addressed *Salvia* taxon monophyly with NGS data. Within *Calosphace* monophyly has been an issue for *S*. sections *sensu* Epling and species, particularly in sections with disjunct distribution and widely distributed and variable species. The discordance between morphological recognition of sections *s*. Epling and later molecular phylogenies have also been discussed elsewhere (Jenks et al., 2013; Fragoso-Martínez et al., 2018) and has been hypothesized to be caused by morphological homoplasy due to pollinator pressure.

Species monophyly has been addressed several times in *S*. subg. *Calosphace* through traditional Sanger sequencing, mostly rejecting monophyly. For example, Walker (2006) sampled several specimens each of *S. polystachia, S. purpurea*, and *S. tiliifolia*, and only the latter was monophyletic in his neighbor-joining tree. Later Jenks et al. (2013) found *S. microphylla, S. mexicana*, and *S. polystachia* to be non-monophyletic. In our results, *S. hispanica* and *S. purpurea* are monophyletic whereas traditional Sanger (Walker, 2006) sequencing rejected *S. purpurea* monophyly. However, our massive alignment was not sufficient to test monophyly for *S. polystachia* nor *S. tiliifolia*. Species monophyly for these and other species will likely need a distinct approach, such as phylogeography (Cutter, 2013), to get a better grasp at the speciation processes, particularly for such morphologically complex and amply distributed species.

In this study, we provide valuable new evidence as to the utility of Hyb-Seq data for capturing 96 nuclear loci from phylogenetically distant Lamiaceae and closely related *Salvia* subg. *Calosphace*, including testing species monophyly. We also recovered the cpDNA genome with concatenated tree phylogeny in agreement with the nuclear genome with this sampling and with previous phylogenies and improved clade resolution. We found two newly supported monophyletic *S*. subg. *Calosphace* sections *s*. Epling and two of four species tested were monophyletic. Although this is the largest NGS study of *Salvia* to date, a more thorough taxon sampling is necessary to better test sectional relationships. NGS-based approaches combined with the reassessment of morphological characters are needed to re-assess sectional circumscription, study the complex species groups in subg. *Calosphace*, and eventually produce a new monograph. Beyond the implications for systematics, a robust phylogeny for the genus is necessary to test hypotheses about the evolution of pollinator associations and morphological adaptations to pollinators. We hope that sage researchers will use our bait design across the width of the phylogenetic spectrum as a steppingstone to build upon for future studies.

## Data Availability Statement

The datasets presented in this study can be found in online repositories. The names of the repository/repositories and accession number(s) can be found below: BioSample: PRJNA748827.

## Author Contributions

GG, JP, and SL-C conceived the study and data analysis. SL-C contributed to the laboratory work. CM-L and MP-G contributed to data analysis. AF, AC-J, and JM-C contributed to analysis and manuscript review. All authors contributed to manuscript writing and review, read, and approved the final manuscript.

## Funding

This work was supported by the following: CONACyT graduate studies scholarship 618610 MP-G, CONACyT sabbatical scholarship 232839 to SL-C, Coordinación de la Investigación Científica (UMSNH), Project 8.16. NSF Award Number 1120080 to JP. And NSF Grant DEB-1557059 that supports CM-L post-doctoral position.

## Conflict of Interest

The authors declare that the research was conducted in the absence of any commercial or financial relationships that could be construed as a potential conflict of interest.

## Publisher's Note

All claims expressed in this article are solely those of the authors and do not necessarily represent those of their affiliated organizations, or those of the publisher, the editors and the reviewers. Any product that may be evaluated in this article, or claim that may be made by its manufacturer, is not guaranteed or endorsed by the publisher.
