# Supplementary material for: Phylogenomics of Salvia L. subgenus Calosphace (Lamiaceae)
Source: Front Plant Sci. 2021 Oct 15;12:725900. doi: 10.3389/fpls.2021.725900 (PMC8554000; doi:10.3389/fpls.2021.725900)
Supplement: Supplementary file 1 [file Table_1.docx]

Supplementary Table 1. Taxa sampled in this study; sectional classifications *sensu* Epling. Including collection data, voucher herbaria, genebank accession and whether the species was sampled in previous phylogenies, number of samples per section is indicated and number of species in each.

| ***Salvia* subg. *Calosphace* Section *s*. Epling** | **Species** | **Locality of collection** | **Voucher** | **Genbank Accession** | **Jenks et al., 2013** | **Fragoso-Martínez et al., 2017** |
| --- | --- | --- | --- | --- | --- | --- |
|  | *Poliomintha incana* (Torr.) A. Gray | *Godden, G. & Ruiz Rubio, N. 116 (112)*. USA. New Mexico, Rio Arriba Santa Fe National Forest. Madera Canyon. Santa Fe National Forest Tailhead 4, accessible from NM Highway 84 between Abiquiu and Hernandez. Approximately 7 miles SE of Abiqui and 3.25 miles from junction with NM 554. 36°10'11.160"N 106°13'12.564" W, 1789 m. 08/23/2011 | FLAS | SAMN20345277 |  |  |
|  | *Cantinoa mutabilis* (Rich.) Harley & J.F.B.Pastore | *Godden, G. 287.* USA. Florida. Alachua, Gainesville. University of Florida Campus. Along drainage ditch and trail south of Bartram Woods. 06/04/2015 | FLAS | SAMN20345278 |  |  |
|  | *Hedeoma drummondii* Benth. | *Godden, G. & Ruiz Rubio, N.* 122. USA. New Mexico. Otero Lincoln National Forest. Sacramento Mountains. Approximately 1 air miles south of Mayhill, along Miller Flats Rd., ca. 0.5 miles from junction with Rio Penasco Road, NM 130. 32°52'34.524" N 105°28'21.972" W, 2039 m. 08/25/2011 | FLAS | SAMN20345279 |  |  |
|  | *Agastache* *pallidiflora ssp. neomexicana* (Briq.) Lint & Epling | *G. Godden, 123.* USA. New Mexico, Grant Gila National Forest. Along Hwy 15, just beyond the McMillan campground on west side of road. 32°55'41.460" N 108°12'38.688" W, 2102 m. 08/30/2011 | FLAS | SAMN20345280 |  |  |
|  | *Dracocephalum parviflorum* Nutt. | *Halse, R.R. 6624.* USA. New Mexico. Rio Arriba Co. Along US Hwy 64. ca. 12.5 miles SW of Dulce. 36° 46’ 53’’N 107° 6’ 19’’W. | RSA | SAMN20345281 |  |  |
|  | *Lepechinia hastata* (A. Gray) Epling | *RSABG, 20809.* USA. California. In cultivation at Rancho Santa Ana Botanic Garden, from cuttings | RSA | SAMN20345282 |  |  |
|  | *Lepechinia* sp. | *Porter, J. M., Prather, L. A., Batallas, R. & Álvarez, O. 14103.* Ecuador. Prov. Azuay, S of the turn-off to Nabon and N of Oña, along Highway 35 (Pan-American Highway), 03°22.867’S, 79°11.255’W, ca, 3,890 m. 05/13/2004 | RSA | SAMN20345283 |  |  |
|  | *Lycopus americanus*  Muhl. | *Wood, 2507.* USA. California. Sacramento river: city of Redding. Small pond open to the river on the W side of the river ± 0.5 miles upstream of Cypress Ave. Bridge. 40° 34’ 40’’N 122° 21’ 12’’W, 143m. 09/01/2010 | RSA | SAMN20345284 |  |  |
|  | *Melissa officinalis* L. | *Lara-Cabrera, S. 176*. USA. California. From live plant bought to "Flowers by the sea". | EBUM | SAMN20345285 |  | + |
|  | *Prunella vulgaris* L. | *Godden, G. & Ruiz Rubio, N. 118.* USA. New Mexico. Otero Lincoln National Forest. Near Mascalero Apache Reservation: Sacramento Mountains. Along NM Hwy 255, near mile marker 7. 33° 00’30.054’’ N, 105°39’12.552’’W, 2388m. 08/25/2011 | FLAS | SAMN20345286 |  |  |
| *Audibertia* (15/2) | *Salvia brandegeei* Munz | *RSABG, 22651.* Mexico. Baja California. In cultivation at RSA from wild material collected in San Quintin. 03/01/2015. | RSA | SAMN20345287 |  |  |
|  | *Salvia sonomensis* Greene | *Hills, G. 22198.* USA. California, RSABG accession in cultivation from wild collected San Juan. 05/17/2007 | RSA | SAMN20345288 |  |  |
| *Salvia* | *Salvia officinalis* L. | *Godden, G. 285.* USA. California. Los Angeles. Claremont, Private residence at 1876 Abilene Way. Cultivated in the garden of Judy Ott-McGoon. 34°07.040'N, 117°42.751’ W, 429 m. 03/19/2015 | RSA | SAMN20345289 |  | + |
|  | *Salvia sessilifolia* Baker | *J.M. Porter 15821*. Cultivated: U.S.A. California, San Bernardino County, Rancho Cucamonga, 6405 Revere Ave. Source: Huntington Botanical Gardens; voucher—*J. Lavranos 11297*; Madagascar, Fianarantsoa, Amoron'i Mania Region, Itremo. | RSA | SAMN20345290 |  |  |
| *Salviastrum* | *Salvia texana* (Scheele) Torr. | *Godden, G. & Ruiz Rubio, N.* 88. USA. Texas, Tarrant Avondale. Along Penden Road, opposite Eagle Mountain Lake State Park, at junction of Farm to Market 718, 32°56'07.650" N 97°28'36.858" W. 08/07/2011 | RSA | SAMN20345291 |  |  |
| *Albolanatae* (1/2) | *Salvia leucantha* Cav. | *Porter, J. M. & Lara-Cabrera, S.* 15318. USA. California. In cultivation at Huntington Botanical Garden. 09/18/2014 | EBUM | SAMN20345292 | + | + |
| *Angulatae* (4/52) | *Salvia tiliifolia* Vahl [15] | *Bedolla-García, B. 15.* Mexico. Guanajuato. Carr. Santa Ana Maya-Iramuco, pasando Puerto Las Cabras, 3.5km al E. Acámbaro, 19°58.39N 100°56.33W, 1824m. 09/21/2008 | EBUM | SAMN20345293 | + | + |
|  | *Salvia tiliifolia* Vahl [5] | *Cibrián, A. 5.* Mexico. Estado de Mexico. Mpio. Valle de Santiago. Parcela La Escuela. 20°17'51.3"N 101°07'52.8"W. 10/24/2014. | LANGEBIO | SAMN20345294 |  | + |
|  | *Salvia longispicata* M. Martens & Galeotti | *Lara-Cabrera, S. 116. Mexico. Guerrero. A 19 km en carr. Chilpancingo a Chihu*alco, a 3km del Palmar. 17°30'39"N 99°37'13"W, 1547m. 11/24/2006 | EBUM | SAMN20345295 | + | + |
|  | *Salvia roscida* Fernald | *Porter, J. M. & Lara-Cabrera, S. 15365*. USA. California in cultivation at University of California Botanic Garden at Berkeley from wild collection. México. Sinaloa. 10/09/2014 | RSA | SAMN20345296 |  |  |
|  | *Salvia rhyacophila* (Fernald) Epling | *Cruz Durán R. s.n.* Mexico. Guerrero. Mpio. General Heliodoro Castillo. Tlacotepec, 12.2km and NO camino a Huautla. 1998. | FCME | SAMN20345297 |  | + |
| *Atratae* (1/1) | *Salvia semiatrata* Zucc. | *Porter, J. M. & Lara-Cabrera, S. 15337*. In cultivation at Cabrillo Botanical Garden, California EUA. 10/01/ 2014 | RSA | SAMN20345298 | + | + |
| *Axillares* (1/1) | *Salvia axillaris* Moc. & Sessé | *Chiang, F. & Azumbilla, A. L. 2625.* Mexico. Puebla. On the road toward Cañada Morelos. 06/09/1985 | RSA | SAMN20345299 | + | + |
| *Biflorae* (2/4) | *Salvia squalens* Kunth | *Porter, J.M. & Columbus, J.T. 12157.* Perú. Department Piura. 40 km E of the intersection of the old Panam Highway 4A/3N to Jean and Moyabamba; steep eroding cliff walls, along side of highway, 05°51’ 02’’S, 79°31’26’’W, 1850 m. 04/11/2000 | RSA | SAMN20345300 |  | + |
|  | *Salvia tubiflora* Sm. | *Porter, J. M. & Lara-Cabrera, S. 15317.* USA. California. In cultivation at Huntington Botanical Garden. 09/18/2014 | RSA | SAMN20345301 | + | + |
| *Blakea* (2/5) | *Salvia patens* Cav. | *Lara-Cabrera, S. 137.* Mexico. Hidalgo. Mpio. Mineral del Chico. Camino a Mineral del Chico. 20.08.42N, 98.41.31W. 09/14/2008 | EBUM | SAMN20345302 | + | + |
|  | *Salvia vitifolia* Benth. | *Porter, J. M. & Lara-Cabrera, S.* 15340. USA. California. In cultivation at Cabrillo Botanic Garden. 10/01/ 2014 | RSA | SAMN20345303 | + | + |
| *Brandegeia* (1/3) | *Salvia blepharophylla* Brandegee | *Porter, J. M. & Lara-Cabrera, S.* 15325, USA. California. In cultivation at Huntington Botanical Garden. 09/18/2014 | RSA | SAMN20345304 | + | + |
| *Briquetia* (1/5) | *Salvia mexicana* L. | *Bedolla-García, B. & Olvera, E. 59.* Mexico, Guerrero. Carretera hacia Ciudad Altamirano. 17°58'51.4'' N 101°12'50.2''W, 1829m. 10/30/2009 | EBUM | SAMN20345305 | + | + |
| *Cardinales* (5/9) | *Salvia karwinskii* Benth. | *Porter, J. M. & Lara-Cabrera, S. 15362.* USA. California. In cultivation at University of California Botanic Garden at Berkeley from wild collected. México. Chiapas. *Ornduff, R. 8194B*. 10/09/2014 | RSA | SAMN20345306 | + | + |
|  | *Salvia wagneriana* Pol. | *Porter, J. M. & Lara-Cabrera, S.* 15354. USA. California. In cultivation at University of California Botanic Garden at Berkeley from wild collected. México. Chiapas. *Breedlove, D.E. 42822*. 10/09/2014 | RSA | SAMN20345307 | + | + |
|  | *Salvia involucrata* Cav. | *Porter, J. M. & Lara-Cabrera, S.* 15320. México. Querétaro. Staff expedition near border with S.L.P., N of Hwy 120W of Xilitla, along road to Pozo y Valle de Guadalupe. 11/17/1971. In cultivation at Huntington Botanical Garden. California, EUA. 09/18/2014 | RSA | SAMN20345308 | + | + |
|  | *Salvia puberula* Fernald | *Porter, J. M. & Lara-Cabrera, S. 15349.* In cultivation at University of California Botanic Garden at Berkeley, from wild collection México. Nuevo León. *Fairey, J. T27M5OS.* 10/02/2014 | RSA | SAMN20345309 |  |  |
|  | *Salvia univerticillata* Ramamoorthy ex Klitg. | *Porter, J. M. & Lara-Cabrera, S. 15355.* USA. California. In cultivation at Green House RSABG, from cuttings. 03/01/2015 | RSA | SAMN20345310 |  | + |
| *Cucullatae* (1/1) | *Salvia clinopodioides* Kunth | *Porter, J. M. & Lara-Cabrera, S. 15336.* USA. California. In cultivation at Cabrillo Botanic Garden. 10/08/2014 | RSA | SAMN20345311 | + | + |
| *Curtiflorae* (3/9) | *Salvia curtiflora* Epling | *Porter, J. M. & Lara-Cabrera, S. 15356.* USA. In cultivation at University of California Botanic Garden at Berkeley, from wild collecion Guatemala. *Wiliamowski, S. 5.* 10/02/2014 | RSA | SAMN20345312 | + |  |
|  | *Salvia nervata* M. Martens & Galeotti | *Bedolla-García, B. & Domínguez, G. 101.* Mpio. Siltepec. Chiapas, México. A las afueras de Siltepec. 15 26 57N, 92, 15, 58W, 2874m. Chiapas. México. 12/28/2009 | EBUM | SAMN20345313 | + | + |
|  | *Salvia longistyla* Benth. | *Porter, J. M. & Lara-Cabrera, S. 15353.* USA. In cultivation at University of California Botanic Garden at Berkeley, from wild collection. México. Nuevo León. *Fairey, J. & C. Schoenfeld T73-119.* 10/09/2014 | RSA | SAMN20345314 | + | + |
| *Dusenostachy*s (2/9) | *Salvia madrensis* Seem. | *Porter, J. M. & Lara-Cabrera, S. 15321.* USA. California. In cultivation at Huntington Botanic Garden. 09/18/2014 | RSA | SAMN20345315 | + | + |
|  | *Salvia divinorum* Epling & Játiva | *Porter, J. M. & Lara-Cabrera, S. 15359.* USA. In cultivation at University of California Botanic Garden at Berkeley, from wild collecion. México. Oaxaca. *Reisfield, S. s.n.* 10/09/2014 | RSA | SAMN20345316 | + | + |
| *Erythrostachys* (1/4) | *Salvia regla* Cav. | *Porter, J. M. & Lara-Cabrera, S. 15328.* USA. California. In cultivation at Huntington Botanic Garden. 09/18/2014 | RSA | SAMN20345317 | + | + |
| *Farinaceae* (2/10) | *Salvia farinacea* Benth. | *Godden, G. & Ruiz Rubio, N. 94.* USA. Texas, Country, Brewster, Near Marathon. Along US Route 90 between Alpine and Marathon; ca. 20 road miles from Alpine, Texas. 30°15'21.240"N, 103°24'13.572" W. 08/12/2011 | FLAS | SAMN20345318 | + | + |
|  | *Salvia azurea* Michx. ex Vahl | *Godden, G. & Ruiz Rubio, N. 111.* USA. New Mexico, Country Taos, Carson National Forest. 36°19'27.53" N, 105°35'07.37"W. 08/22/2011 | FLAS | SAMN20345319 | + | + |
| *Flexuosae* (1/4) | *Salvia pauciserrata* Benth. | *Godden, G. et al 279.* Colombia. Cundinamarca. Along main road between Caparrapí and La Palma. 05°23.394’ N, 074°27.522’ W. WGS84. Elev. 1136 m. 02/04/2015. | COL | SAMN20345320 |  | + |
| *Flocculosae* (3/18) | *Salvia chamaedryoides* Cav. | *Breedlove, D. E. & D. Mahoney 72154*. México. Nuevo León. In cultivation at University of California Botanic Garden at Berkeley. 10/02/2014 | RSA | SAMN20345321 | + | + |
|  | *Salvia coahuilensis* Fernald | *Porter, J. M. & Lara-Cabrera, S.* 15329. USA. California. In cultivation at Cabrillo Botanic Garden. 10/08/2014 | RSA | SAMN20345322 | + | + |
|  | *Salvia greggii* A. Gray | *Porter, J. M. & Lara-Cabrera, S. 15315*. USA. California. In cultivation at Huntington Botanic Garden. 09/18/2014 | RSA | SAMN20345323 | + | + |
| *Fulgentes* (3/6) | *Salvia fulgens* Cav. | *Lara-Cabrera, S. 143.* México. Estado de Mexico. Carretera México-Toluca, La Marquesa. 09/16/2008 | EBUM | SAMN20345324 | + | + |
|  | *Salvia dichlamys* Epling | *Lara-Cabrera, S. 175.* USA. California. Cultivated material bought to "Flowers by the sea". 03/15/2015 | RSA | SAMN20345325 |  |  |
|  | *Salvia microphylla* Kunth | *Porter, J. M. & Lara-Cabrera, S.* 15327. Mexico. Hidalgo. La Placita d., 85, km 165. In cultivation at Huntington Botanic Garden. California. USA. 09/18/2014 | RSA | SAMN20345326 | + | + |
| *Hastatae* (2/7) | *Salvia macrophylla* Benth. | *Porter, J. M. 12255* USA. California. Rancho Santa Ana Botanic Garden. Cultivated material, seeds collected near Chachapoyas, Dept. Amazonas. Peru. 06/30/2000 | RSA | SAMN20345327 | + | + |
|  | *Salvia scutellarioides* Kunth | *Godden, G. et al. 275.* Colombia. Cundinamarca. Along side road to San Francisco, off main route from El Rosal to El Chuscal. 04°54.825’ N, 074°17.469’ W, WGS84 Elev. 1958 m. 02/01/2015 | COL | SAMN20345328 | + | + |
| *Incarnatae* (2/2) | *Salvia elegans* Vahl | *Lara-Cabrera, S. 136.* Mexico. Hidalgo, Mpio, Mineral del Chico, Camino a Mineral del Chico. 20.08.24N 98.41.31W. 2170m. 09/14/2008 | EBUM | SAMN20345329 | + | + |
|  | *Salvia cinnabarina* M. Martens & Galeotti | *Bedolla-García, B. & Domínguez, G. 102.* Mpio. Siltepec. Chiapas, México. A las afueras de Siltepec. 15 26 57N, 92, 15, 58W, 2874m. Chiapas. México. 12/28/2009 | EBUM | SAMN20345330 | + | + |
| *Iodanthae* (1/1) | *Salvia iodantha* Fernald | *Bedolla-García, B. 66.* Mexico. Michoacan. Cerro Patamban, entrando por Aranza. 12/31/2008 | EBUM | SAMN20345331 | + | + |
| *Lavanduloideae* (2/18) | *Salvia helianthemifolia* Benth. | *Bedolla-García, B. 28.* Mexico. Guanajuato. Victoria, sobre la brecha, pasando Higueras. 21°16'14.8"N 100°08'08.6"W 2456msnm. 11/04/2008 | EBUM | SAMN20345332 |  | + |
|  | *Salvia lavanduloides* Kunth | *Lara-Cabrera, S. 155.* Mexico. Morelos. Carretera Tepoztlán San Juan Tlacotenco 19.00.08N; 99.06.99W. 31/10/2009 | EBUM | SAMN20345333 | + | + |
| *Maxoniae* (1/7) | *Salvia chiapensis* Fernald | *Porter, J. M. & Lara-Cabrera, S. 15364.* USA. California. In cultivation at University of California Botanic Garden at Berkeley from wild collected. México. Chiapas. *Bartholomew, B. & D. E. Breedlove 965*. 10/09/2014 | RSA | SAMN20345334 | + | + |
| *Mitratae* (1/2) | *Salvia lasiantha* Benth. | *Veliz, M. 24375.* Guatemala. Huehutenango, Chiantla. La Zeta. 15°21'40.24'' N 91° 26' 12'' W, 2184 m. 09/23/ 2014 | EBUM | SAMN20345335 | + | + |
| *Nobiles* (2/12) | *Salvia disjuncta* Fernald | *Veliz, M. & Olvera, E. 24376*. Guatemala, Huehuetenago, Chiantla, cerca del Mirador, 09/23/2014 | EBUM | SAMN20345336 | + | + |
|  | *Salvia gesneriiflora* Lindl. & Paxton | *Lara-Cabrera, S. 112.* Mexico. Estado de Mexico. Carr. Huitzilac a Lagunas de Zempoala en el marcaje km 10. 19° 18' 23'' N, 99° 13' 44''W. 2924 m. 11/04/2006 | EBUM | SAMN20345337 | + | + |
| *Polystachyae* (9/16) | *Salvia polystachia* Ortega [163] | *Lara-Cabrera, S. 163.* Mexico. Morelos, 5km al S de Tetela del Volcán camino a Temoac. 18° 50’ 55’’N, 98° 44’ 16’’W, 2030m. 11/15/2009 | EBUM | SAMN20345338 | + | + |
|  | *Salvia polystachia* Ortega [65] | *Bedolla-García, B. 65.* Mexico. Michoacan. En los alrededores de Tlalpujahua Mpio. Tlalpujahua. 19° 48’ 05’’ N 100° 11’ 40’’ W, 2633m. 11/14/2009 | EBUM | SAMN20345339 | + | + |
|  | *Salvia brachyodonta* Briq. | *Bedolla-García, B. 51.* Mexico. Jalisco. Parque mirador independencia. Guadalajara. 20° 44' 25'' N 103° 18' 36.1'' W, 1509m. 10/17/2009 | EBUM | SAMN20345340 |  |  |
|  | *Salvia decora* Epling | *Calonico-Soto J. & Blanco, A. 27187*. Mexico. Guerrero. Primer campo, 5.56km al NE del Aguacate. Coahuayutla, 18°12'00.5" N 101°24'58"W, 2113m. 11/02/2007 | EBUM | SAMN20345341 |  |  |
|  | *Salvia filipes* Benth. | *Lara-Cabrera, S. 161.* Mexico. Hidalgo. Camino de los Prismas Basálticos a Ex-Haciencda Santa María Regla, a 50 m de Recinas. 20°14’15’’N, 98°33’49’’W, 2020m. 11/14/2009 | EBUM | SAMN20345342 |  | + |
|  | *Salvia perblanda* Epling | *Calonico-Soto J. & Blanco, A. 27191*. Mexico. Guerrero. Primer campo, 5.56km al NE del Aguacate. Coahuayutla, 18º12'00.5" N 101º24'58"W, 2113m. 11/02/2007 | EBUM | SAMN20345343 |  |  |
|  | *Salvia plurispicata* Epling | *Bedolla-García, B. 64.* Mexico. Michoacan.Cerro Patamban entrando por Aranza. 12/31/2008 | EBUM | SAMN20345344 |  | + |
|  | *Salvia connivens* Epling | *Olvera, E. 4.* Mexico. Guanajuato. Mpio. Xichú en llano grande a unos metros donde comienza el río. 06/10/2008 | EBUM | SAMN20345345 |  | + |
|  | *Salvia purepecha* Bedolla, S. Lara Cabrera & Zamudio | *Bedolla-García, B. 52.* Mexico. Michoacan. Mpio. Tangancícuaro, a las afueras de Patamban, sobre la carretera Patamban-Aranza, a la orilla de un río estacional. 12/31/2009 | EBUM | SAMN20345346 |  |  |
|  | *Salvia tonaticensis* Ramamoorthy ex Lara-Cabrera, Bedolla & Zamudio | *Zamudio, S.* *et al.* *15242*. Mexico. Estado de Mexico, In cultivation from wild collection at Barranca de Tonatico. 18°47'43''N, 99°,42'49'' W, 1420 m. | EBUM | SAMN20345347 |  | + |
| *Potiles* (1/1) | *Salvia hispanica* L. [85] | *Tenorio, P & Dieringer, D. 10685*. Mexico, Oaxaca Teotitlán de Flores Magón, Cerro Verde, 23 Km. al NE de Teotitlán, carr. Teotitlán-Huautla. 12/10/1985. | MEXU | SAMN20345348 |  | + |
|  | *Salvia hispanica* L. [19] | *Gonzalez, s.n.* Mexico. Guerrero. Mpio. Leonardo Bravo. La Laguna, 6km adelante del poblado. 17°43’ 54’’N, 99°44’40’’W, 1997. | FCME | SAMN20345349 |  | + |
| *Purpureae* (3/9) | *Salvia curviflora* Benth. | *Bedolla-García, 71.* Mexico. Guanajuato. Piedras de Lúmbre, Jerécuaro. 20°14.46N, 100°.36.34 W, 2352m. 11/16/2009 | EBUM | SAMN20345350 |  | + |
|  | *Salvia purpurea* Cav. [103] | *Bedolla-García, B. 103.* Mexico. Chiapas. A las afueras de Siltepec. Mpio. Siltepec. 15° 26’ 57’’N, 92° 15’ 58’’W. 2874m. 12/28/2009 | EBUM | SAMN20345351 | + | + |
|  | *Salvia purpurea* Cav. [156] | *Lara-Cabrera, S. 156.* Mexico. Morelos. Carretera Tepoztlán San Juan Tlacotenco, 19°.00.08N; 99°.06.99W. 10/31/2009 | EBUM | SAMN20345352 |  | + |
|  | *Salvia areolata* Epling | *Veliz, M. & Olvera, E. 24377*. Guatemala, Huehuetenango, Chiantla, cerca del Mirador. 15° 23 57 N 91° 26 20.5W, 2981m. 09/23/2014 | EBUM | SAMN20345353 | + | + |
| *Secundae* (1/10) | *Salvia splendens* Sellow ex Wied-Neuw. | *Lara-Cabrera, S. 174.* USA. California. Cultivated material bought to "Flowers by the sea". 03/15/2015 | RSA | SAMN20345354 | + | + |
| *Scorodoniae* (7/16) | *Salvia aequidistans* Fernald | *Olvera, E. 22A.* Mexico. Nayarit. Camino de Compostela hacia Tepic entre 11 y 13 kms. 10/30/2014 | EBUM | SAMN20345355 |  | + |
|  | *Salvia breviflora* Moc. & Sessé | *Lara-Cabrera, S. 171.* Mexico. Morelos. 3.7 km al S en desviación a Tlayca de la carretera Cuautla-Izucar de Matamoros, Mex 160. 11/02/2013 | EBUM | SAMN20345356 |  | + |
|  | *Salvia keerlii* Benth. | *Olvera, E. & J. Olvera-Sanchez. J. 10.* Mexico. Queretaro. Mpio. Colón, camino entre Bernal y Santiago de Queretaro. 11/02/2013 | EBUM | SAMN20345357 | + | + |
|  | *Salvia melissodora* Lag. | *Olvera, E. & J. Olvera-Sanchez. J. 25.* Mexico. Hidalgo. Ixmiquilpan. 11/04/2013 | EBUM | SAMN20345358 | + | + |
|  | *Salvia occidua* Epling | *Olvera, E. 1.* Mexico. Nayarit. Distrito Pochutla en San Miguel del Puerto, 12/18/2012 | EBUM | SAMN20345359 |  |  |
|  | *Salvia ramosa* Brandegee | *Montero, J.C. 1548.* Mexico. Oaxaca. Carretera Tlaxiaco-Putla, 9.5 Km by SW de Tlaxiaco, chapel of the Virgin de Guadalupe. Distrito of Tlaxiaco. 09/14/2008 | EBUM | SAMN20345360 | + | + |
|  | *Salvia dugesii* Fernald | *Olvera, E. & Olvera, J. 5.* Mexico. Guanajuato. Mpio. San Miguel de Allende, Charco del Ingenio Botanical Garden. 09/18/2013 | EBUM | SAMN20345361 |  |  |
| *Sigmoideae* (2/11) | *Salvia inconspicua* Bertol. | *Montero, J. C. 1555.* Mexico. Oaxaca. Juxtlahuaca carretea a San Juan Piñas, 8km al W de San Sebastián Tecomatlahuaca. 09/14/2013 | EBUM | SAMN20345362 | + |  |
|  | *Salvia nepetoides* Kunth | *Lara-Cabrera S., 115a.* Mexico. Guerrero. A 19 km en carr. Chilpancingo a Chihualco, a 3km del Palmar. 17°30'39"N 99°37'13"W, 1547m. 11/24/2006 | EBUM | SAMN20345363 |  |  |
| *Standleyana* (1/1) | *Salvia cacaliifolia* Benth. | *Veliz, M. 24378.* Guatemala, Huehuetenango, Chiantla, La Capellina, 15 24 31N 91 25 55W, 3158m. 11/23/2014 | EBUM | SAMN20345364 | + | + |
| *Uricae* (2/2) | *Salvia amarissima* Ortega | *Olvera, E. & Olvera J. 16.* Mexico. Querétaro. Peña de Bernal. going up to the Peña, Mpio Ezequiel Montes. 09/18/2013 | EBUM | SAMN20345365 | + | + |
|  | *Salvia urica* Epling | *Veliz, M. 24364.* Guatemala, Sacatepéquez, San Lucas Sac., recta del Mirador a San Lucas, 14° 36 04N 91° 38 36W, 2096m. 11/30/2014 | EBUM | SAMN20345366 | + | + |
