# Supplementary material for: Phylogenomics of Salvia L. subgenus Calosphace (Lamiaceae)
Source: Front Plant Sci. 2021 Oct 15;12:725900. doi: 10.3389/fpls.2021.725900 (PMC8554000; doi:10.3389/fpls.2021.725900)
Supplement: Supplementary file 2 [file Table_2.docx]

Supplementary Table 2. Transcriptomes from One Thousand Plant Transcriptomes Initiative (2019) and GenBank used with MarkerMiner 1.0 (Chamala et al., 2015) to identify and select low copy orthologous genes for this study.

| **Species** | **Order** | **Family** | **ID** | **Reference** |
| --- | --- | --- | --- | --- |
| *Ehretia acuminata* R. Br. | Boraginales | Boraginaceae | EMAL | One Thousand Plant Transcriptomes Initiative |
| *Lennoa madreporoides* La Llave & Lex. | Boraginales | Boraginaceae | SMUR | One Thousand Plant Transcriptomes Initiative |
| *Mertensia paniculata* (Aiton) G.Don. | Boraginales | Boraginaceae | DKFZ | One Thousand Plant Transcriptomes Initiative |
| *Phacelia campanularia* A.Gray | Boraginales | Boraginaceae | YQIJ | One Thousand Plant Transcriptomes Initiative |
| *Pholisma arenarium* Nutt. | Boraginales | Boraginaceae | HANM | One Thousand Plant Transcriptomes Initiative |
| *Exacum affine* Balf. f. | Gentianales | Gentianaceae | KPUM | One Thousand Plant Transcriptomes Initiative |
| *Galium boreale* L. | Gentianales | Rubiaceae | WQRD | One Thousand Plant Transcriptomes Initiative |
| *Anisacanthus quadrifidus* Standl. | Lamiales | Acanthaceae | PCGJ | One Thousand Plant Transcriptomes Initiative |
| *Ruellia brittoniana* Leonard | Lamiales | Acanthaceae | AYIY | One Thousand Plant Transcriptomes Initiative |
| *Sanchezia* Ruiz & Pav. *sp*. | Lamiales | Acanthaceae | NBMW | One Thousand Plant Transcriptomes Initiative |
| *Strobilanthes dyeriana* Mast. | Lamiales | Acanthaceae | WEAC | One Thousand Plant Transcriptomes Initiative |
| *Kigelia africana* (Lam.) Benth. | Lamiales | Bignoniaceae | QKEI | One Thousand Plant Transcriptomes Initiative |
| *Kigelia africana* (Lam.) Benth. | Lamiales | Bignoniaceae | SVQC | One Thousand Plant Transcriptomes Initiative |
| *Mansoa alliacea* (Lam.) A. H. Gentry | Lamiales | Bignoniaceae | TKEK | One Thousand Plant Transcriptomes Initiative |
| *Tabebuia umbellata* (Sond.) Sandwith | Lamiales | Bignoniaceae | UTQR | One Thousand Plant Transcriptomes Initiative |
| *Byblis gigantea* Lindl. | Lamiales | Byblidaceae | GDZS | One Thousand Plant Transcriptomes Initiative |
| *Calceolaria pinifolia* Cav. | Lamiales | Calceolariaceae | DCCI | One Thousand Plant Transcriptomes Initiative |
| *Saintpaulia ionantha* H. Wendl. | Lamiales | Gesneriaceae | RWKR | One Thousand Plant Transcriptomes Initiative |
| *Sinningia tuberosa* (Mart.) H. E. Moore | Lamiales | Gesneriaceae | DTNC | One Thousand Plant Transcriptomes Initiative |
| *Agastache rugosa* Kuntze. | Lamiales | Lamiaceae | PUCW | One Thousand Plant Transcriptomes Initiative |
| *Ajuga reptans* L. | Lamiales | Lamiaceae | UCNM | One Thousand Plant Transcriptomes Initiative |
| *Clinopodium serpyllifolium*  subsp. *fruticosum* (L.) Bräuchler | Lamiales | Lamiaceae | WHNV | One Thousand Plant Transcriptomes Initiative |
| *Coleus scutellarioides* (L.) Benth. | Lamiales | Lamiaceae | BAHE | One Thousand Plant Transcriptomes Initiative |
| *Lavandula angustifolia* Mill. | Lamiales | Lamiaceae | FYUH | One Thousand Plant Transcriptomes Initiative |
| *Leonurus japonicus* Houtt. | Lamiales | Lamiaceae | SNNC | One Thousand Plant Transcriptomes Initiative |
| *Marrubium vulgare* L. | Lamiales | Lamiaceae | EAAA | One Thousand Plant Transcriptomes Initiative |
| *Melissa officinalis* L. | Lamiales | Lamiaceae | TAGM | One Thousand Plant Transcriptomes Initiative |
| *Nepeta cataria* L. | Lamiales | Lamiaceae | FUMQ | One Thousand Plant Transcriptomes Initiative |
| *Oxera neriifolia* (Montrouz.) Beauvis. | Lamiales | Lamiaceae | GNPX | One Thousand Plant Transcriptomes Initiative |
| *Oxera pulchella* Labill. | Lamiales | Lamiaceae | RTNA | One Thousand Plant Transcriptomes Initiative |
| *Pogostemon cablin* (Blanco) Benth. | Lamiales | Lamiaceae | GETL | One Thousand Plant Transcriptomes Initiative |
| *Poliomintha bustamanta* B. L. Turner | Lamiales | Lamiaceae | XMBA | One Thousand Plant Transcriptomes Initiative |
| *Prunella vulgaris* L. | Lamiales | Lamiaceae | PHCE | One Thousand Plant Transcriptomes Initiative |
| *Salvia officinalis* L. | Lamiales | Lamiaceae | EQDA | One Thousand Plant Transcriptomes Initiative |
| *Salvia splendens* Sellow ex Wied-Neuw. | Lamiales | Lamiaceae | 180765 | (Ge et al. 2015) GenBank |
| *Scutellaria montana* Chapm. | Lamiales | Lamiaceae | ATYL | One Thousand Plant Transcriptomes Initiative |
| *Rosmarinus officinalis* L*.* | Lamiales | Lamiaceae | FDMM | One Thousand Plant Transcriptomes Initiative |
| *Teucrium chamaedrys* L. | Lamiales | Lamiaceae | LRRR | One Thousand Plant Transcriptomes Initiative |
| *Thymus vulgaris* L. | Lamiales | Lamiaceae | IYDF | One Thousand Plant Transcriptomes Initiative |
| *Pinguicula agnata* Casper | Lamiales | Lentibulariaceae | MXFG | One Thousand Plant Transcriptomes Initiative |
| *Pinguicula caudata* Schltdl. | Lamiales | Lentibulariaceae | JCMU | One Thousand Plant Transcriptomes Initiative |
| *Utricularia* L. *sp*. | Lamiales | Lentibulariaceae | HRUR | One Thousand Plant Transcriptomes Initiative |
| *Chionanthus retusus* Paxton | Lamiales | Oleaceae | KTAR | One Thousand Plant Transcriptomes Initiative |
| *Forestiera segregata* (Jacq.) Krug & Urb. | Lamiales | Oleaceae | UEEN | One Thousand Plant Transcriptomes Initiative |
| *Ligustrum sinense* Lour. | Lamiales | Oleaceae | MZLD | One Thousand Plant Transcriptomes Initiative |
| *Olea europaea* L. | Lamiales | Oleaceae | TORX | One Thousand Plant Transcriptomes Initiative |
| *Conopholis americana* (L.) Wallr. | Lamiales | Orobanchaceae | FAMO | One Thousand Plant Transcriptomes Initiative |
| *Epifagus virginiana* (L.) W.P.C.Barton | Lamiales | Orobanchaceae | URZI | One Thousand Plant Transcriptomes Initiative |
| *Epifagus virginiana* (L.) W. P. C. Barton | Lamiales | Orobanchaceae | XMOG | One Thousand Plant Transcriptomes Initiative |
| *Lindenbergia philippinensis* Benth. | Lamiales | Orobanchaceae | WUZV | One Thousand Plant Transcriptomes Initiative |
| *Lindenbergia philippinensis* Benth. | Lamiales | Orobanchaceae | ZVFS | One Thousand Plant Transcriptomes Initiative |
| *Orobanche fasciculata* Nutt. | Lamiales | Orobanchaceae | PHOQ | One Thousand Plant Transcriptomes Initiative |
| *Orobanche fasciculata* Nutt. | Lamiales | Orobanchaceae | VTOK | One Thousand Plant Transcriptomes Initiative |
| *Paulownia fargesii* Franch. | Lamiales | Paulowniaceae | UMUL | One Thousand Plant Transcriptomes Initiative |
| *Uncarina grandidieri* (Beaill.) Stapf | Lamiales | Pedaliaceae | ZRIN | One Thousand Plant Transcriptomes Initiative |
| *Rehmannia glutinosa* Steud. | Lamiales | Rhemanniaceae | OWAS | One Thousand Plant Transcriptomes Initiative |
| *Antirrhinum majus* L. | Lamiales | Plantaginaceae | EBOL | One Thousand Plant Transcriptomes Initiative |
| *Antirrhinum majus* L. | Lamiales | Plantaginaceae | TPUT | One Thousand Plant Transcriptomes Initiative |
| *Antirrhinum braun-blanquetii* Rothm. | Lamiales | Plantaginaceae | YRHD | One Thousand Plant Transcriptomes Initiative |
| *Bacopa caroliniana* (Walter) B. L. Rob. | Lamiales | Plantaginaceae | CLRW | One Thousand Plant Transcriptomes Initiative |
| *Digitalis purpurea* L. | Lamiales | Plantaginaceae | GNRI | One Thousand Plant Transcriptomes Initiative |
| *Plantago coronopus* L. | Lamiales | Plantaginaceae | DCVZ | One Thousand Plant Transcriptomes Initiative |
| *Plantago maritima* L. | Lamiales | Plantaginaceae | YKZB | One Thousand Plant Transcriptomes Initiative |
| *Plantago virginica* L. | Lamiales | Plantaginaceae | PTBJ | One Thousand Plant Transcriptomes Initiative |
| *Schlegelia parasitica* Griseb. | Lamiales | Schlegeliaceae | GAKQ | One Thousand Plant Transcriptomes Initiative |
| *Schlegelia parasitica* Griseb. | Lamiales | Schlegeliaceae | CWLL | One Thousand Plant Transcriptomes Initiative |
| *Schlegelia violacea* Griseb. | Lamiales | Schlegeliaceae | EDXZ | One Thousand Plant Transcriptomes Initiative |
| *Anticharis glandulosa* Asch. | Lamiales | Scrophulariaceae | EJBY | One Thousand Plant Transcriptomes Initiative |
| *Buddleja* L. *sp*. | Lamiales | Scrophulariaceae | GRFT | One Thousand Plant Transcriptomes Initiative |
| *Buddleja lindleyana* Lindl. | Lamiales | Scrophulariaceae | XRLM | One Thousand Plant Transcriptomes Initiative |
| *Celsia arcturus* Jacq. | Lamiales | Scrophulariaceae | SIBR | One Thousand Plant Transcriptomes Initiative |
| *Verbascum* L. *sp.* | Lamiales | Scrophulariaceae | XXYA | One Thousand Plant Transcriptomes Initiative |
| *Polypremum procumbens* L. | Lamiales | Tetrachondraceae | COBX | One Thousand Plant Transcriptomes Initiative |
| *Lantana camara* L. | Lamiales | Verbenaceae | PSHB | One Thousand Plant Transcriptomes Initiative |
| *Phyla dulcis* (Trev.) Moldenke | Lamiales | Verbenaceae | MQIV | One Thousand Plant Transcriptomes Initiative |
| *Verbena hastata* L. | Lamiales | Verbenaceae | GCFE | One Thousand Plant Transcriptomes Initiative |
| *Ipomoea pubescens* Lam. | Solanales | Convolvulaceae | EMBR | One Thousand Plant Transcriptomes Initiative |
| *Solanum ptychanthum* Dunal | Solanales | Solanaceae | DLJZ | One Thousand Plant Transcriptomes Initiative |
