# Supplementary material for: Phylogenomics of Salvia L. subgenus Calosphace (Lamiaceae)
Source: Front Plant Sci. 2021 Oct 15;12:725900. doi: 10.3389/fpls.2021.725900 (PMC8554000; doi:10.3389/fpls.2021.725900)
Supplement: Supplementary file 3 [file Table_3.docx]

Supplementary Table 3. Loci selection based on the *Arabidopsis thaliana* annotated genome. Loci names include the *Arabidopsis thaliana* gene and whether the baits were designed with *Salvia* or Lamiales-wide reference sequences.

| Name | % Pairwise Identity | # Sequences | Max Sequence Length | Min Sequence Length | Sequence Length | Estimated No Baits |
| --- | --- | --- | --- | --- | --- | --- |
| AT1G05120_Salvia | 0.632 | 2 | 4596 | 4590 | 4609 | 40 |
| AT1G07010_Salvia | 0.632 | 2 | 1888 | 1781 | 1901 | 18 |
| AT1G11545_Salvia | 0.821 | 2 | 942 | 931 | 946 | 10 |
| AT1G16970_Salvia | 0.53 | 2 | 4521 | 4520 | 4526 | 40 |
| AT1G35190_Salvia | 0.505 | 2 | 2130 | 2090 | 2187 | 20 |
| AT1G51940_Salvia | 0.695 | 2 | 2872 | 2842 | 2882 | 26 |
| AT2G04305_Salvia | 0.707 | 2 | 1824 | 1806 | 1824 | 17 |
| AT2G36895_Salvia | 0.675 | 2 | 1158 | 1155 | 1158 | 12 |
| AT2G45770_Salvia | 0.577 | 2 | 2101 | 2076 | 2170 | 20 |
| AT2G45990_Salvia | 0.589 | 2 | 1644 | 1644 | 1644 | 16 |
| AT3G01720_Salvia | 0.694 | 2 | 3280 | 2851 | 3288 | 29 |
| AT3G04260_Salvia | 0.673 | 2 | 4301 | 4249 | 4301 | 38 |
| AT3G04480_Salvia | 0.672 | 2 | 3406 | 3382 | 3406 | 30 |
| AT3G09030_Salvia | 0.866 | 2 | 1413 | 1387 | 1422 | 14 |
| AT3G11830_Salvia | 0.59 | 2 | 3429 | 3429 | 3432 | 31 |
| AT3G12290_Salvia | 0.716 | 2 | 1304 | 1301 | 1306 | 13 |
| AT3G17040_Salvia | 0.642 | 2 | 3128 | 3125 | 3131 | 28 |
| AT3G17940_Salvia | 0.676 | 2 | 1626 | 1610 | 1629 | 16 |
| AT3G22590_Salvia | 0.821 | 2 | 1353 | 1351 | 1354 | 13 |
| AT3G23620_Salvia | 0.621 | 2 | 1737 | 1734 | 1737 | 17 |
| AT3G25660_Salvia | 0.644 | 2 | 2654 | 2331 | 2750 | 25 |
| AT3G25900_Salvia | 0.615 | 2 | 1736 | 1734 | 1736 | 17 |
| AT3G27530_Salvia | 0.537 | 2 | 5891 | 5882 | 5900 | 51 |
| AT3G29010_Salvia | 0.715 | 2 | 1110 | 1109 | 1115 | 11 |
| AT3G43540_Salvia | 0.51 | 2 | 2337 | 2158 | 2348 | 22 |
| AT3G46610_Salvia | 0.881 | 2 | 1807 | 1662 | 1813 | 17 |
| AT3G46790_Salvia | 0.789 | 2 | 1867 | 1412 | 1947 | 18 |
| AT3G48460_Salvia | 0.64 | 2 | 1695 | 1680 | 1711 | 16 |
| AT3G48610_Salvia | 0.624 | 2 | 2336 | 2313 | 2352 | 22 |
| AT3G52640_Salvia | 0.507 | 2 | 5164 | 5042 | 5167 | 45 |
| AT3G55260_Salvia | 0.562 | 2 | 3581 | 3567 | 3602 | 32 |
| AT3G56460_Salvia | 0.694 | 2 | 1465 | 1453 | 1469 | 14 |
| AT3G57790_Salvia | 0.796 | 2 | 1731 | 1519 | 1734 | 17 |
| AT3G58690_Salvia | 0.665 | 2 | 1884 | 1865 | 1887 | 18 |
| AT3G59040_Salvia | 0.71 | 2 | 2435 | 2416 | 2445 | 22 |
| AT3G59380_Salvia | 0.659 | 2 | 1651 | 1637 | 1658 | 16 |
| AT3G60830_Salvia | 0.662 | 2 | 1697 | 1644 | 1700 | 16 |
| AT3G60850_Salvia | 0.937 | 2 | 1971 | 1627 | 1998 | 19 |
| AT4G01880_Salvia | 0.663 | 2 | 2117 | 2099 | 2126 | 20 |
| AT4G02990_Salvia | 0.893 | 2 | 1566 | 1560 | 1569 | 15 |
| AT4G04930_Salvia | 0.622 | 2 | 1692 | 1632 | 1692 | 16 |
| AT4G09750_Salvia | 0.567 | 2 | 1929 | 1929 | 1929 | 18 |
| AT4G19490_Salvia | 0.558 | 2 | 7094 | 7079 | 7143 | 62 |
| AT4G20130_Salvia | 0.866 | 2 | 1743 | 1615 | 1796 | 17 |
| AT4G30510_Salvia | 0.875 | 2 | 1221 | 930 | 1259 | 13 |
| AT4G35870_Salvia | 0.91 | 2 | 2648 | 2565 | 2806 | 25 |
| AT4G38460_Salvia | 0.776 | 2 | 1286 | 1271 | 1301 | 13 |
| AT5G05660_Salvia | 0.646 | 2 | 4155 | 3757 | 4166 | 37 |
| AT5G14720_Salvia | 0.559 | 2 | 4412 | 4409 | 4412 | 39 |
| AT5G18070_Salvia | 0.867 | 2 | 1704 | 1674 | 1704 | 16 |
| AT5G50390_Salvia | 0.872 | 2 | 2112 | 1721 | 2186 | 20 |
| AT5G56580_Salvia | 0.66 | 2 | 1767 | 1764 | 1767 | 17 |
| AT5G61530_Salvia | 0.641 | 2 | 1934 | 1931 | 1934 | 18 |
| AT5G63610_Salvia | 0.942 | 2 | 1419 | 1416 | 1419 | 14 |
| AT1G05350_E1_Lamiales | 0.866 | 57 | 155 | 145 | 155 | 3 |
| AT1G14300_E1_Lamiales | 0.667 | 53 | 416 | 368 | 416 | 6 |
| AT1G14300_E2_Lamiales | 0.84 | 53 | 146 | 132 | 146 | 3 |
| AT1G14300_E3_Lamiales | 0.812 | 53 | 246 | 235 | 360 | 5 |
| AT1G14810_E1_Lamiales | 0.795 | 52 | 484 | 288 | 503 | 6 |
| AT1G14810_E2_Lamiales | 0.806 | 52 | 155 | 47 | 155 | 3 |
| AT1G28340_E1_Lamiales | 0.662 | 55 | 1552 | 1289 | 1559 | 15 |
| AT1G28340_E2_Lamiales | 0.659 | 55 | 398 | 350 | 410 | 6 |
| AT1G43860_E1_Lamiales | 0.499 | 64 | 1812 | 644 | 1834 | 17 |
| AT1G53280_E1_Lamiales | 0.669 | 68 | 730 | 126 | 741 | 8 |
| AT1G53280_E2_Lamiales | 0.64 | 68 | 778 | 201 | 778 | 9 |
| AT1G62750_Lamiales | 0.841 | 63 | 2085 | 1679 | 2131 | 20 |
| AT1G64550_E1_Lamiales | 0.589 | 67 | 1866 | 830 | 1913 | 18 |
| AT1G64550_E2_Lamiales | 0.822 | 67 | 147 | 147 | 147 | 3 |
| AT1G64550_E3_Lamiales | 0.79 | 63 | 120 | 120 | 120 | 3 |
| AT2G15230_E1_Lamiales | 0.839 | 55 | 143 | 42 | 143 | 3 |
| AT2G15230_E2_Lamiales | 0.836 | 55 | 144 | 122 | 144 | 3 |
| AT2G15230_E3_Lamiales | 0.79 | 55 | 301 | 183 | 301 | 5 |
| AT2G18710_Lamiales | 0.731 | 57 | 1559 | 1286 | 1569 | 15 |
| AT2G18940_Lamiales | 0.793 | 63 | 1861 | 669 | 1885 | 18 |
| AT2G19940_E1_Lamiales | 0.835 | 52 | 120 | 120 | 120 | 3 |
| AT2G19940_E2_Lamiales | 0.662 | 50 | 326 | 8 | 326 | 5 |
| AT2G31880_E1_Lamiales | 0.708 | 60 | 421 | 138 | 421 | 6 |
| AT2G31880_E2_Lamiales | 0.737 | 60 | 1095 | 799 | 1149 | 12 |
| AT2G37500_E1_Lamiales | 0.87 | 48 | 124 | 41 | 124 | 3 |
| AT2G37500_E2_Lamiales | 0.844 | 53 | 156 | 123 | 156 | 3 |
| AT2G37500_E3_Lamiales | 0.823 | 53 | 171 | 23 | 171 | 4 |
| AT3G05350_E1_Lamiales | 0.697 | 54 | 545 | 540 | 545 | 7 |
| AT3G05350_E2_Lamiales | 0.567 | 54 | 1683 | 1067 | 1683 | 16 |
| AT3G06510_E2_Lamiales | 0.61 | 56 | 2122 | 1185 | 2124 | 20 |
| AT3G09180_E1_Lamiales | 0.714 | 55 | 505 | 250 | 507 | 6 |
| AT3G09180_E2_Lamiales | 0.772 | 55 | 348 | 318 | 357 | 5 |
| AT3G10230_Lamiales | 0.754 | 60 | 1370 | 992 | 1402 | 14 |
| AT3G17810_Lamiales | 0.604 | 56 | 1873 | 1737 | 1882 | 18 |
| AT3G20790_E1_Lamiales | 0.627 | 57 | 367 | 137 | 367 | 5 |
| AT3G20790_E2_Lamiales | 0.781 | 57 | 131 | 105 | 131 | 3 |
| AT3G45300_E1_Lamiales | 0.855 | 59 | 611 | 310 | 653 | 8 |
| AT3G45300_E2_Lamiales | 0.86 | 59 | 155 | 140 | 155 | 3 |
| AT3G45300_E3_Lamiales | 0.831 | 57 | 147 | 47 | 147 | 3 |
| AT3G47610_E1_Lamiales | 0.586 | 56 | 1277 | 1138 | 1331 | 13 |
| AT3G47610_E2_Lamiales | 0.776 | 56 | 224 | 206 | 230 | 4 |
| AT3G51050_E1_Lamiales | 0.545 | 59 | 917 | 390 | 917 | 10 |
| AT3G51050_E2_Lamiales | 0.722 | 60 | 441 | 404 | 454 | 6 |
| AT3G51050_E3_Lamiales | 0.75 | 60 | 852 | 668 | 860 | 9 |
| AT3G52190_E1_Lamiales | 0.793 | 54 | 395 | 147 | 408 | 5 |
| AT3G52190_E2_Lamiales | 0.671 | 54 | 336 | 280 | 339 | 5 |
| AT3G52190_E3_Lamiales | 0.721 | 53 | 177 | 100 | 189 | 4 |
| AT3G53700_Lamiales | 0.783 | 56 | 1859 | 807 | 1877 | 18 |
| AT3G55070_E1_Lamiales | 0.531 | 56 | 2181 | 1885 | 2215 | 21 |
| AT3G56460_E1_Lamiales | 0.789 | 55 | 413 | 168 | 419 | 6 |
| AT3G56460_E2_Lamiales | 0.703 | 55 | 497 | 496 | 497 | 6 |
| AT3G58460_E2_Lamiales | 0.674 | 54 | 174 | 123 | 177 | 4 |
| AT3G66658_E1_Lamiales | 0.674 | 55 | 601 | 353 | 601 | 7 |
| AT3G66658_E2_Lamiales | 0.571 | 55 | 1513 | 914 | 1520 | 15 |
| AT4G00090_E1_Lamiales | 0.629 | 53 | 644 | 591 | 651 | 8 |
| AT4G00090_E2_Lamiales | 0.833 | 50 | 204 | 204 | 204 | 4 |
| AT4G00740_E1_Lamiales | 0.683 | 55 | 1927 | 1533 | 1967 | 18 |
| AT4G19860_E1_Lamiales | 0.807 | 55 | 175 | 99 | 175 | 4 |
| AT4G19860_E2_Lamiales | 0.823 | 57 | 136 | 131 | 136 | 3 |
| AT4G19860_E3_Lamiales | 0.649 | 57 | 448 | 420 | 454 | 6 |
| AT4G19860_E4_Lamiales | 0.781 | 54 | 266 | 162 | 266 | 4 |
| AT4G29490_E1_Lamiales | 0.525 | 65 | 900 | 881 | 906 | 10 |
| AT4G29490_E2_Lamiales | 0.837 | 64 | 159 | 57 | 159 | 3 |
| AT4G29830_Lamiales | 0.611 | 58 | 1486 | 1206 | 1506 | 15 |
| AT4G30310_E1_Lamiales | 0.67 | 57 | 423 | 134 | 423 | 6 |
| AT4G30310_E2_Lamiales | 0.625 | 57 | 799 | 766 | 821 | 9 |
| AT4G30310_E3_Lamiales | 0.669 | 55 | 337 | 128 | 337 | 5 |
| AT4G31990_E1_Lamiales | 0.843 | 57 | 152 | 150 | 152 | 3 |
| AT4G31990_E2_Lamiales | 0.616 | 57 | 1340 | 963 | 1340 | 13 |
| AT4G35850_E1_Lamiales | 0.596 | 57 | 486 | 440 | 507 | 6 |
| AT4G35850_E2_Lamiales | 0.81 | 61 | 210 | 201 | 210 | 4 |
| AT4G37040_E1_Lamiales | 0.735 | 66 | 480 | 229 | 482 | 6 |
| AT4G37040_E2_Lamiales | 0.819 | 66 | 194 | 79 | 194 | 4 |
| AT5G04420_E1_Lamiales | 0.661 | 58 | 382 | 367 | 382 | 5 |
| AT5G04420_E2_Lamiales | 0.764 | 58 | 327 | 303 | 339 | 5 |
| AT5G05200_E1_Lamiales | 0.843 | 65 | 151 | 5 | 151 | 3 |
| AT5G05200_E2_Lamiales | 0.73 | 65 | 776 | 764 | 778 | 9 |
| AT5G05200_E3_Lamiales | 0.831 | 65 | 179 | 37 | 179 | 4 |
| AT5G05200_E4_Lamiales | 0.863 | 64 | 130 | 98 | 130 | 3 |
| AT5G06260_E1_Lamiales | 0.693 | 58 | 404 | 404 | 404 | 5 |
| AT5G08100_E1_Lamiales | 0.627 | 55 | 607 | 234 | 607 | 7 |
| AT5G08100_E2_Lamiales | 0.775 | 55 | 375 | 357 | 375 | 5 |
| AT5G08170_E1_Lamiales | 0.678 | 57 | 695 | 490 | 695 | 8 |
| AT5G09860_E1_Lamiales | 0.879 | 52 | 146 | 101 | 146 | 3 |
| AT5G09860_E2_Lamiales | 0.851 | 54 | 126 | 126 | 126 | 3 |
| AT5G13030_E1_Lamiales | 0.652 | 56 | 2040 | 1961 | 2061 | 19 |
| AT5G13520_Lamiales | 0.666 | 53 | 2474 | 1956 | 2544 | 23 |
| AT5G13650_E1_Lamiales | 0.696 | 57 | 743 | 395 | 744 | 8 |
| AT5G13650_E2_Lamiales | 0.814 | 57 | 135 | 134 | 135 | 3 |
| AT5G13650_E3_Lamiales | 0.861 | 57 | 152 | 152 | 152 | 3 |
| AT5G13650_E4_Lamiales | 0.65 | 57 | 708 | 453 | 708 | 8 |
| AT5G14250_E1_Lamiales | 0.552 | 62 | 498 | 236 | 502 | 6 |
| AT5G14250_E2_Lamiales | 0.802 | 63 | 224 | 201 | 224 | 4 |
| AT5G14250_E3_Lamiales | 0.709 | 62 | 384 | 103 | 384 | 5 |
| AT5G17530_E1_Lamiales | 0.845 | 55 | 186 | 165 | 186 | 4 |
| AT5G17530_E2_Lamiales | 0.802 | 55 | 237 | 216 | 237 | 4 |
| AT5G17530_E3_Lamiales | 0.833 | 54 | 155 | 152 | 155 | 3 |
| AT5G19540_E1_Lamiales | 0.642 | 58 | 1367 | 1300 | 1398 | 14 |
| AT5G30510_E1_Lamiales | 0.677 | 58 | 586 | 243 | 586 | 7 |
| AT5G30510_E2_Lamiales | 0.85 | 58 | 282 | 264 | 282 | 4 |
| AT5G42310_E1_Lamiales | 0.787 | 58 | 1564 | 608 | 1591 | 15 |
| AT5G42480_E1_Lamiales | 0.715 | 57 | 930 | 465 | 943 | 10 |
| AT5G42480_E2_Lamiales | 0.71 | 57 | 519 | 405 | 519 | 6 |
| AT5G43600_E1_Lamiales | 0.827 | 53 | 131 | 123 | 131 | 3 |
| AT5G43600_E2_Lamiales | 0.795 | 53 | 203 | 191 | 203 | 4 |
| AT5G43600_E3_Lamiales | 0.806 | 53 | 241 | 241 | 241 | 4 |
| AT5G46580_Lamiales | 0.779 | 62 | 1869 | 1173 | 1938 | 18 |
| AT5G57030_E1_Lamiales | 0.725 | 55 | 371 | 136 | 371 | 5 |
| AT5G57030_E2_Lamiales | 0.837 | 55 | 144 | 136 | 144 | 3 |
| AT5G57030_E3_Lamiales | 0.652 | 54 | 612 | 352 | 624 | 7 |
| AT5G57655_E1_Lamiales | 0.854 | 64 | 130 | 130 | 130 | 3 |
| AT5G57655_E2_Lamiales | 0.772 | 65 | 603 | 587 | 604 | 7 |
| AT5G57655_E3_Lamiales | 0.802 | 64 | 152 | 44 | 152 | 3 |
| AT5G62530_E1_Lamiales | 0.81 | 61 | 212 | 209 | 213 | 4 |
| AT5G62530_E2_Lamiales | 0.796 | 58 | 139 | 127 | 139 | 3 |
| AT5G63890_E1_Lamiales | 0.75 | 61 | 630 | 387 | 631 | 7 |
| AT5G64370_E1_Lamiales | 0.715 | 66 | 1270 | 991 | 1270 | 13 |
| AT5G65720_Lamiales | 0.826 | 60 | 1262 | 952 | 1264 | 13 |
| General purpose_Lamiales | 87.151 |  | 75442 | 51910 | 76472 | 883 |
| Salvia_specific | 37.398 |  | 130464 | 126257 | 131394 | 1207 |
| Total | 124.549 |  | 205906 | 178167 | 207866 | 2091 |
