# Supplementary material for: Phylogenomics of Salvia L. subgenus Calosphace (Lamiaceae)
Source: Front Plant Sci. 2021 Oct 15;12:725900. doi: 10.3389/fpls.2021.725900 (PMC8554000; doi:10.3389/fpls.2021.725900)
Supplement: Supplementary file 4 [file Table_4.docx]

Supplementary Table 4. Assembly report for HPM. Loci captured for all 90 samples are shaded in a gray background and the assembly with lowest sample capture and highest in bold. Alignment length in bold font are longest (AT4G19490) and shortest alignments (AT1G053590). 54 general purpose targeted low copy nuclear genes are underlined and 42 *Salvia* designed baits are not underlined, arranged in ascending order for the number of taxa recovered for each locus.

| **Locus name** | **# samples** | **Targeted alignment (bp)** | **Alignment (bp)** | **# targeted exons** | **Avg. exons recovered** | **Missing %** | **# variable sites** | **Proportion variable sites** | **Parsimony informative sites** | **Proportion parsimony informative** | **AT (%)** | **GC (%)** |
| --- | --- | --- | --- | --- | --- | --- | --- | --- | --- | --- | --- | --- |
| AT1G05120 | 88 | 2700 | 2714 | 16 | 11.01 | 25.168 | 819 | 0.302 | 504 | 0.186 | 57.8 | 42.2 |
| AT1G05350 | 90 | **153** | **154** | 1 | 1 | 1.219 | 49 | 0.318 | 24 | 0.156 | 57.8 | 42.2 |
| AT1G07010 | 89 | 1073 | 1200 | 10 | 5.25 | 44.096 | 262 | 0.218 | 162 | 0.135 | 57.8 | 42.2 |
| AT1G14300 | 89 | 703 | 833 | 3 | 2.88 | 21.215 | 259 | 0.311 | 146 | 0.175 | 57.8 | 42.2 |
| AT1G14810 | 89 | 567 | 658 | 2 | 1.91 | 17.532 | 179 | 0.272 | 123 | 0.187 | 57.8 | 42.2 |
| AT1G16970 | 86 | 1931 | 1937 | 17 | 8.33 | 44.224 | 411 | 0.212 | 207 | 0.107 | 57.8 | 42.2 |
| AT1G28340 | 89 | 1511 | 1544 | 2 | 1.9 | 4.868 | 560 | 0.363 | 347 | 0.225 | 57.8 | 42.2 |
| AT1G43860 | 90 | 464 | 851 | 1 | 1 | 45.942 | 166 | 0.195 | 115 | 0.135 | 57.8 | 42.2 |
| AT1G51940 | 88 | 1946 | 1986 | 11 | 7.81 | 26.923 | 612 | 0.308 | 409 | 0.206 | 57.8 | 42.2 |
| AT1G53280 | 90 | 1074 | 1091 | 2 | 1.99 | 2.998 | 425 | 0.39 | 262 | 0.24 | 57.8 | 42.2 |
| AT1G62750 | 90 | 2066 | 2130 | 1 | 1 | 4.255 | 594 | 0.279 | 401 | 0.188 | 57.8 | 42.2 |
| AT1G64550 | 90 | 1377 | 1450 | 3 | 2.79 | 11.549 | 442 | 0.305 | 283 | 0.195 | 57.8 | 42.2 |
| AT2G04305 | 69 | 1341 | 1369 | 4 | 2.52 | 21.256 | 337 | 0.246 | 238 | 0.174 | 57.8 | 42.2 |
| AT2G15230 | 90 | 586 | 587 | 3 | 2.52 | 25.955 | 191 | 0.325 | 123 | 0.21 | 57.8 | 42.2 |
| AT2G18710 | 90 | 1274 | 1294 | 4 | 3.37 | 13.947 | 409 | 0.316 | 277 | 0.214 | 57.8 | 42.2 |
| AT2G19940 | 89 | 357 | 357 | 2 | 1.91 | 5.143 | 118 | 0.331 | 78 | 0.218 | 57.8 | 42.2 |
| AT2G36895 | 85 | 735 | 738 | 5 | 2.7 | 39.396 | 180 | 0.244 | 105 | 0.142 | 57.8 | 42.2 |
| AT2G37500 | 90 | 393 | 447 | 3 | 2.93 | 16.348 | 148 | 0.331 | 92 | 0.206 | 57.8 | 42.2 |
| AT3G01720 | 87 | 2078 | 2515 | 8 | 5.9 | 43.193 | 640 | 0.254 | 366 | 0.146 | 57.8 | 42.2 |
| AT3G04260 | 90 | 2752 | 2753 | 13 | 9.39 | 21.545 | 848 | 0.308 | 482 | 0.175 | 57.8 | 42.2 |
| AT3G04480 | 85 | 2236 | 2236 | 12 | 4.33 | 49.404 | 662 | 0.296 | 382 | 0.171 | 57.8 | 42.2 |
| AT3G05350 | 90 | 1343 | 1351 | 2 | 2 | 6.746 | 457 | 0.338 | 289 | 0.214 | 57.8 | 42.2 |
| AT3G09180 | 90 | 725 | 769 | 2 | 1.27 | 41.64 | 212 | 0.276 | 113 | 0.147 | 57.8 | 42.2 |
| AT3G10230 | 77 | 1348 | 1402 | 1 | 0.85 | 5.796 | 364 | 0.26 | 256 | 0.183 | 57.8 | 42.2 |
| AT3G11830 | 90 | 1680 | 1683 | 14 | 10 | 18.4 | 450 | 0.267 | 275 | 0.163 | 57.8 | 42.2 |
| AT3G12290 | 89 | 923 | 928 | 4 | 3.71 | 9.522 | 292 | 0.315 | 204 | 0.22 | 57.8 | 42.2 |
| AT3G17040 | 87 | 1857 | 1863 | 8 | 6.64 | 22.136 | 555 | 0.298 | 335 | 0.18 | 57.8 | 42.2 |
| AT3G17810 | 90 | 1138 | 1149 | 7 | 6.4 | 9.251 | 325 | 0.283 | 230 | 0.2 | 57.8 | 42.2 |
| AT3G17940 | 86 | 1114 | 1119 | 5 | 3.99 | 13.895 | 359 | 0.321 | 216 | 0.193 | 57.8 | 42.2 |
| AT3G20790 | 89 | 391 | 398 | 2 | 1.96 | 7.413 | 152 | 0.382 | 99 | 0.249 | 57.8 | 42.2 |
| AT3G22590 | 89 | 1145 | 1148 | 3 | 1.29 | 15.587 | 332 | 0.289 | 218 | 0.19 | 57.8 | 42.2 |
| AT3G23620 | 90 | 954 | 957 | 6 | 5.48 | 8.964 | 305 | 0.319 | 204 | 0.213 | 57.8 | 42.2 |
| AT3G25660 | 90 | 1541 | 1959 | 9 | 6.73 | 41.037 | 454 | 0.232 | 232 | 0.118 | 57.8 | 42.2 |
| AT3G25900 | 82 | 1001 | 1001 | 6 | 3.64 | 32.336 | 307 | 0.307 | 194 | 0.194 | 57.8 | 42.2 |
| AT3G27530 | 89 | 2757 | 2770 | 19 | 5.87 | 46.089 | 704 | 0.254 | 403 | 0.145 | 57.8 | 42.2 |
| AT3G29010 | 90 | 869 | 874 | 4 | 2.26 | 51.584 | 255 | 0.292 | 127 | 0.145 | 57.8 | 42.2 |
| AT3G43540 | 83 | 1053 | 1064 | 8 | 3.73 | 51.113 | 289 | 0.272 | 165 | 0.155 | 57.8 | 42.2 |
| AT3G45300 | 90 | 722 | 954 | 3 | 2.9 | 27.249 | 215 | 0.225 | 132 | 0.138 | 57.8 | 42.2 |
| AT3G47610 | 87 | 1013 | 1110 | 2 | 1.42 | 25.905 | 342 | 0.308 | 200 | 0.18 | 57.8 | 42.2 |
| AT3G51050 | 90 | 1613 | 1681 | 3 | 2.96 | 7.866 | 499 | 0.297 | 319 | 0.19 | 57.8 | 42.2 |
| AT3G52190 | 89 | 725 | 849 | 3 | 2.92 | 24.951 | 217 | 0.256 | 137 | 0.161 | 57.8 | 42.2 |
| AT3G52640 | 88 | 2069 | 2072 | 15 | 7.03 | 39.467 | 598 | 0.289 | 340 | 0.164 | 57.8 | 42.2 |
| AT3G55070 | 90 | 1051 | 1146 | 1 | 1 | 10.765 | 367 | 0.32 | 237 | 0.207 | 57.8 | 42.2 |
| AT3G55260 | 87 | 1665 | 1686 | 15 | 7.3 | 33.933 | 427 | 0.253 | 260 | 0.154 | 57.8 | 42.2 |
| AT3G55500 | 72 | 383 | 383 | 2 | 0.81 | 46.392 | 78 | 0.204 | 48 | 0.125 | 57.8 | 42.2 |
| AT3G56460 | 88 | 810 | 1120 | 7 | 5.82 | 23.514 | 366 | 0.327 | 220 | 0.196 | 57.8 | 42.2 |
| AT3G57790 | 87 | 1292 | 1507 | 3 | 2.52 | 30.292 | 453 | 0.301 | 278 | 0.184 | 57.8 | 42.2 |
| AT3G58460 | 84 | 165 | 177 | 1 | 0.93 | 6.04 | 62 | 0.35 | 34 | 0.192 | 57.8 | 42.2 |
| AT3G58690 | 89 | 1220 | 1223 | 6 | 5.01 | 19.348 | 375 | 0.307 | 245 | 0.2 | 57.8 | 42.2 |
| AT3G59040 | 86 | 1788 | 1803 | 5 | 3.9 | 36.226 | 550 | 0.305 | 332 | 0.184 | 57.8 | 42.2 |
| AT3G59380 | 85 | 1060 | 1067 | 5 | 3.07 | 32.447 | 356 | 0.334 | 224 | 0.21 | 57.8 | 42.2 |
| AT3G60830 | 90 | 1033 | 1089 | 5 | 4.8 | 12.687 | 334 | 0.307 | 211 | 0.194 | 57.8 | 42.2 |
| AT3G60850 | 90 | 1971 | 2009 | 1 | 1 | 4.483 | 691 | 0.344 | 457 | 0.227 | 57.8 | 42.2 |
| AT3G66658 | 90 | 1219 | 1236 | 2 | 2 | 2.819 | 373 | 0.302 | 235 | 0.19 | 57.8 | 42.2 |
| AT4G00090 | 89 | 637 | 647 | 2 | 1.92 | 3.895 | 228 | 0.352 | 154 | 0.238 | 57.8 | 42.2 |
| AT4G00740 | 90 | 1319 | 1354 | 1 | 1 | 4.687 | 400 | 0.295 | 261 | 0.193 | 57.8 | 42.2 |
| AT4G01880 | 81 | 1390 | 1417 | 9 | 5.34 | 48.851 | 442 | 0.312 | 241 | 0.17 | 57.8 | 42.2 |
| AT4G02990 | 86 | 1566 | 1655 | 1 | 0.96 | 13.228 | 650 | 0.393 | 436 | 0.263 | 57.8 | 42.2 |
| AT4G09750 | 85 | 969 | 969 | 10 | 5.21 | 32.752 | 248 | 0.256 | 157 | 0.162 | 57.8 | 42.2 |
| AT4G19490 | 90 | **3282** | **3336** | 18 | 11.93 | 21.718 | 1072 | 0.321 | 668 | 0.2 | 57.8 | 42.2 |
| AT4G19860 | 89 | 883 | 927 | 4 | 3.79 | 11.785 | 282 | 0.304 | 180 | 0.194 | 57.8 | 42.2 |
| AT4G20130 | 90 | 1615 | 1796 | 1 | 1 | 20.795 | 592 | 0.33 | 356 | 0.198 | 57.8 | 42.2 |
| AT4G29490 | 90 | 554 | 594 | 2 | 1.99 | 10.619 | 204 | 0.343 | 116 | 0.195 | 57.8 | 42.2 |
| AT4G29830 | 89 | 972 | 1042 | 2 | 1.54 | 34.721 | 275 | 0.264 | 189 | 0.181 | 57.8 | 42.2 |
| AT4G30310 | 89 | 1081 | 1109 | 3 | 2.92 | 8.007 | 315 | 0.284 | 207 | 0.187 | 57.8 | 42.2 |
| AT4G30510 | 90 | 930 | 1258 | 1 | 1 | 37.303 | 345 | 0.274 | 194 | 0.154 | 57.8 | 42.2 |
| AT4G31990 | 90 | 961 | 961 | 2 | 1.99 | 3.539 | 298 | 0.31 | 192 | 0.2 | 57.8 | 42.2 |
| AT4G35850 | 88 | 509 | 545 | 2 | 1.94 | 16.656 | 151 | 0.277 | 94 | 0.172 | 57.8 | 42.2 |
| AT4G35870 | 88 | 2648 | 2806 | 1 | 0.98 | 9.195 | 961 | 0.342 | 624 | 0.222 | 57.8 | 42.2 |
| AT4G37040 | 90 | 585 | 588 | 2 | 1.98 | 9.131 | 200 | 0.34 | 123 | 0.209 | 57.8 | 42.2 |
| AT5G04420 | 90 | 588 | 607 | 2 | 1.89 | 15.457 | 200 | 0.329 | 119 | 0.196 | 57.8 | 42.2 |
| AT5G05200 | 90 | 1063 | 1082 | 4 | 3.1 | 16.537 | 363 | 0.335 | 202 | 0.187 | 57.8 | 42.2 |
| AT5G05660 | 88 | 2664 | 2676 | 12 | 9.17 | 21.942 | 889 | 0.332 | 565 | 0.211 | 57.8 | 42.2 |
| AT5G08170 | 87 | 524 | 527 | 1 | 0.97 | 1.243 | 181 | 0.343 | 122 | 0.231 | 57.8 | 42.2 |
| AT5G09860 | 90 | 272 | 272 | 2 | 2 | 0.09 | 70 | 0.257 | 49 | 0.18 | 57.8 | 42.2 |
| AT5G13030 | 89 | 1396 | 1423 | 1 | 0.99 | 3.773 | 431 | 0.303 | 281 | 0.197 | 57.8 | 42.2 |
| AT5G13520 | 89 | 2058 | 2242 | 6 | 4.64 | 34.135 | 655 | 0.292 | 413 | 0.184 | 57.8 | 42.2 |
| AT5G13650 | 90 | 1309 | 1316 | 4 | 3.89 | 4.07 | 362 | 0.275 | 237 | 0.18 | 57.8 | 42.2 |
| AT5G14250 | 90 | 790 | 836 | 3 | 2.96 | 9.389 | 280 | 0.335 | 167 | 0.2 | 57.8 | 42.2 |
| AT5G14720 | 90 | 2022 | 2025 | 20 | 11.93 | 29.627 | 493 | 0.243 | 300 | 0.148 | 57.8 | 42.2 |
| AT5G17530 | 90 | 576 | 578 | 3 | 2.97 | 2.603 | 220 | 0.381 | 128 | 0.221 | 57.8 | 42.2 |
| AT5G18070 | 86 | 1704 | 1704 | 1 | 0.96 | 7.498 | 614 | 0.36 | 371 | 0.218 | 57.8 | 42.2 |
| AT5G30510 | 90 | 722 | 737 | 2 | 2 | 5.242 | 224 | 0.304 | 158 | 0.214 | 57.8 | 42.2 |
| AT5G42310 | 81 | 1463 | 1505 | 1 | 0.9 | 3.33 | 463 | 0.308 | 327 | 0.217 | 57.8 | 42.2 |
| AT5G42480 | 90 | 1040 | 1052 | 2 | 2 | 2.999 | 390 | 0.371 | 238 | 0.226 | 57.8 | 42.2 |
| AT5G43600 | 89 | 570 | 575 | 3 | 2.37 | 19.541 | 193 | 0.336 | 120 | 0.209 | 57.8 | 42.2 |
| AT5G50390 | 85 | 1635 | 2100 | 2 | 1.87 | 22.838 | 534 | 0.254 | 327 | 0.156 | 57.8 | 42.2 |
| AT5G56580 | 90 | 1068 | 1068 | 8 | 4.08 | 30.12 | 244 | 0.228 | 145 | 0.136 | 57.8 | 42.2 |
| AT5G57030 | 90 | 847 | 889 | 3 | 2.99 | 8.986 | 281 | 0.316 | 176 | 0.198 | 57.8 | 42.2 |
| AT5G57655 | 90 | 806 | 809 | 3 | 2.79 | 11.165 | 252 | 0.311 | 168 | 0.208 | 57.8 | 42.2 |
| AT5G61530 | 87 | 1138 | 1138 | 9 | 4.96 | 29.009 | 355 | 0.312 | 193 | 0.17 | 57.8 | 42.2 |
| AT5G62530 | 79 | 349 | 352 | 2 | 1.61 | 9.555 | 100 | 0.284 | 71 | 0.202 | 57.8 | 42.2 |
| AT5G63610 | 90 | 1416 | 1419 | 1 | 1 | 1.22 | 436 | 0.307 | 273 | 0.192 | 57.8 | 42.2 |
| AT5G63890 | 89 | 535 | 543 | 1 | 0.99 | 2.628 | 174 | 0.32 | 118 | 0.217 | 57.8 | 42.2 |
| AT5G64370 | 90 | 998 | 1004 | 1 | 1 | 3.7 | 372 | 0.371 | 266 | 0.265 | 57.8 | 42.2 |
| AT5G65720 | 88 | 1235 | 1264 | 1 | 0.98 | 4.04 | 378 | 0.299 | 283 | 0.224 | 57.8 | 42.2 |
| **Average** | 87 | 1204.6 | 1261.9 | 4.8 | 3.4 | 19.2 | 377.5 | 0.3 | 235.5 | 0.2 | 57.8 | 42.2 |
