# Supplementary material for: Phylogenomics of Salvia L. subgenus Calosphace (Lamiaceae)
Source: Front Plant Sci. 2021 Oct 15;12:725900. doi: 10.3389/fpls.2021.725900 (PMC8554000; doi:10.3389/fpls.2021.725900)
Supplement: Supplementary file 5 [file Table_5.docx]

Supplementary Table 5. Chloroplast loci from HPM. Genic region and location within the chloroplast plastome are shown.

| **Genic region** | **Location** | **# samples** | **Alingnment (pb)** | **Total matrix cells** | **Missing %** | **Proportion parsimony informative** | **AT (%)** | **GC (%)** | **A (bp)** | **C (bp)** | **G (bp)** | **T (bp)** |
| --- | --- | --- | --- | --- | --- | --- | --- | --- | --- | --- | --- | --- |
| *trn*H_GUG_tRNA | LSC | 90 | 74 | 6660 | 0.631 | 0.027 | 41.9 | 58.1 | 1165 | 1930 | 2240 | 1280 |
| *psb*A_CDS | LSC | 90 | 1059 | 95310 | 0.364 | 0.036 | 56.8 | 43.2 | 22236 | 20566 | 20483 | 31678 |
| *mat*K_CDS | LSC | 88 | 1587 | 139656 | 19.166 | 0.104 | 6.6 | 3.4 | 34620 | 19259 | 18964 | 40047 |
| *trn*K_UUU_tRNA_a | LSC | 87 | 2627 | 228549 | 16.268 | 0.089 | 65.6 | 34.4 | 62122 | 31395 | 34360 | 63492 |
| *rps*16_intron | LSC | 89 | 1113 | 99057 | 6.926 | 0.086 | 64.1 | 35.9 | 30785 | 15194 | 17833 | 28384 |
| *trn*Q_UUG_tRNA | LSC | 89 | 72 | 6408 | 0.312 | 0.111 | 38.9 | 61.1 | 969 | 1683 | 2223 | 1513 |
| *psb*K_CDS | LSC | 89 | 180 | 16020 | 0.094 | 0.05 | 63.4 | 36.6 | 3111 | 3016 | 2837 | 7041 |
| *psb*I_CDS | LSC | 75 | 111 | 8325 | 3.964 | 0.027 | 63.3 | 36.7 | 1792 | 1502 | 1431 | 3270 |
| *trn*S_GCU_tRNA | LSC | 90 | 88 | 7920 | 2.222 | 0.011 | 4.9 | 5.1 | 1595 | 1660 | 2292 | 2197 |
| *trn*G_UCC_tRNA | LSC | 90 | 753 | 67770 | 6.92 | 0.084 | 64.6 | 35.4 | 20651 | 10673 | 11554 | 20202 |
| *trn*R_UCU_tRNA | LSC | 85 | 72 | 6120 | 4.232 | 0 | 56.9 | 43.1 | 1550 | 1136 | 1387 | 1788 |
| *atp*A_CDS | LSC | 90 | 1524 | 137160 | 1.477 | 0.043 | 5.9 | 4.1 | 41806 | 25894 | 29478 | 37956 |
| *atp*F_intron | LSC | 90 | 1263 | 113670 | 5.294 | 0.059 | 64.5 | 35.5 | 35537 | 15823 | 22425 | 33867 |
| *atp*H_CDS | LSC | 89 | 246 | 21894 | 0.005 | 0.028 | 54.5 | 45.5 | 4646 | 3912 | 6045 | 7290 |
| *atp*I_CDS | LSC | 90 | 744 | 66960 | 0.927 | 0.058 | 62.3 | 37.7 | 17712 | 11861 | 13134 | 23632 |
| *rps*2_CDS | LSC | 90 | 711 | 63990 | 0.752 | 0.041 | 59.6 | 40.4 | 20281 | 11243 | 14410 | 17575 |
| *rpo*C2_CDS | LSC | 90 | 4229 | 380610 | 3.917 | 0.064 | 62.1 | 37.9 | 119397 | 64611 | 74050 | 107643 |
| *rpo*C1_CDS_a | LSC | 90 | 2849 | 256410 | 2.551 | 0.046 | 61.9 | 38.1 | 78519 | 42251 | 52917 | 76181 |
| *rpo*B_CDS | LSC | 90 | 3213 | 289170 | 1.127 | 0.044 | 60.8 | 39.2 | 90671 | 48084 | 63968 | 83187 |
| *trn*C_GCA_tRNA | LSC | 90 | 71 | 6390 | 0.125 | 0.014 | 38.2 | 61.8 | 1432 | 1432 | 1785 | 1700 |
| *pet*N_CDS | LSC | 90 | 90 | 8100 | 0.481 | 0.033 | 57.6 | 42.4 | 1684 | 1182 | 2235 | 2960 |
| *psb*M_CDS | LSC | 83 | 105 | 8715 | 4.039 | 0.019 | 68.5 | 31.5 | 2606 | 1674 | 954 | 3129 |
| *trn*D_GUC_tRNA | LSC | 90 | 74 | 6660 | 0.676 | 0.027 | 3.7 | 6.3 | 1165 | 1930 | 2240 | 1280 |
| *trn*Y_GUA_tRNA | LSC | 90 | 84 | 7560 | 0 | 0 | 45.2 | 54.8 | 1620 | 1800 | 2340 | 1800 |
| *trn*E_UUC_tRNA | LSC | 89 | 73 | 6497 | 0 | 0 | 43.8 | 56.2 | 1156 | 1692 | 1958 | 1691 |
| *trn*T_GGU_tRNA | LSC | 88 | 72 | 6336 | 1.026 | 0 | 52.8 | 47.2 | 1744 | 1303 | 1656 | 1568 |
| *psb*D_CDS | LSC | 90 | 1062 | 95580 | 0.247 | 0.034 | 57.8 | 42.2 | 21013 | 18620 | 21560 | 34151 |
| *psb*C_CDS | LSC | 90 | 1422 | 127980 | 0.16 | 0.037 | 56.8 | 43.2 | 30778 | 24269 | 30895 | 41833 |
| *trn*S_UGA_tRNA | LSC | 90 | 93 | 8370 | 0.155 | 0.011 | 50.6 | 49.4 | 2071 | 1881 | 2249 | 2156 |
| *psb*Z_CDS | LSC | 90 | 189 | 17010 | 0.012 | 0.042 | 62.6 | 37.4 | 3419 | 3205 | 3150 | 7234 |
| *trn*G_GCC_tRNA | LSC | 90 | 71 | 6390 | 0.642 | 0 | 49.4 | 50.6 | 1080 | 1784 | 2158 | 1360 |
| *trn*fM_CAU_tRNA | LSC | 90 | 74 | 6660 | 0 | 0 | 44.6 | 55.4 | 1441 | 1710 | 1979 | 1530 |
| *rps*14_CDS | LSC | 90 | 303 | 27270 | 0.092 | 0.04 | 57.6 | 42.4 | 9295 | 5107 | 6448 | 6395 |
| *psa*B_CDS | LSC | 90 | 2205 | 198450 | 0.174 | 0.038 | 58.8 | 41.2 | 49859 | 38383 | 43247 | 66616 |
| *psa*A_CDS | LSC | 90 | 2253 | 202770 | 0.06 | 0.034 | 56.9 | 43.1 | 51214 | 41214 | 46075 | 64145 |
| *ycf*3_CDS_b | LSC | 90 | 1954 | 175860 | 6.352 | 0.058 | 63.3 | 36.7 | 55540 | 27021 | 33396 | 48732 |
| *trn*S_GGA_tRNA | LSC | 90 | 87 | 7830 | 0.741 | 0.034 | 47.4 | 52.6 | 1352 | 1603 | 2485 | 2332 |
| *rps*4_CDS | LSC | 90 | 606 | 54540 | 1.481 | 0.045 | 60.8 | 39.2 | 17863 | 10689 | 10371 | 14809 |
| *trn*T_UGU_tRNA | LSC | 87 | 73 | 6351 | 1.543 | 0 | 46.7 | 53.3 | 1200 | 1536 | 1800 | 1717 |
| *trn*L_UAA_intron | LSC | 90 | 540 | 48600 | 2.07 | 0.089 | 63.2 | 36.8 | 16960 | 8199 | 9306 | 13129 |
| *trn*F_GAA_tRNA | LSC | 90 | 73 | 6570 | 0 | 0 | 49.3 | 50.7 | 1710 | 1530 | 1800 | 1530 |
| *ndh*J_CDS | LSC | 90 | 477 | 42930 | 1.351 | 0.04 | 60.1 | 39.9 | 11927 | 7192 | 9718 | 13513 |
| *ndh*K_CDS | LSC | 90 | 678 | 61020 | 1.136 | 0.058 | 61.7 | 38.3 | 18651 | 11882 | 11223 | 18571 |
| *ndh*C_trnV_IGS | LSC | 90 | 363 | 32670 | 0.594 | 0.047 | 65.4 | 34.6 | 7591 | 4128 | 7097 | 13660 |
| *trn*V_UAC_tRNA | LSC | 90 | 653 | 58770 | 3.737 | 0.08 | 61.6 | 38.4 | 17869 | 10871 | 10854 | 16980 |
| *trn*M_CAU_tRNA | LSC | 90 | 73 | 6570 | 0.502 | 0.068 | 58.8 | 41.2 | 1791 | 1172 | 1518 | 2056 |
| *atp*E_CDS | LSC | 90 | 402 | 36180 | 0.763 | 0.03 | 6.1 | 3.9 | 12120 | 5532 | 8452 | 9800 |
| *atp*B_CDS | LSC | 90 | 1497 | 134730 | 0.706 | 0.043 | 57.5 | 42.5 | 38703 | 25219 | 31578 | 38279 |
| *rbc*L_CDS | LSC | 90 | 1434 | 129060 | 0.034 | 0.05 | 56.5 | 43.5 | 35947 | 24666 | 31492 | 36911 |
| *acc*D_CDS | LSC | 90 | 1494 | 134460 | 6.082 | 0.083 | 62.2 | 37.8 | 38797 | 19376 | 28307 | 39802 |
| *psa*I_CDS | LSC | 86 | 111 | 9546 | 3.457 | 0.018 | 64.8 | 35.2 | 2229 | 1906 | 1333 | 3748 |
| *ycf*4_CDS | LSC | 90 | 555 | 49950 | 0.573 | 0.052 | 6 | 4 | 12210 | 8152 | 11722 | 17580 |
| *cem*A_CDS | LSC | 90 | 691 | 62190 | 5.049 | 0.055 | 67.5 | 32.5 | 18096 | 9697 | 9450 | 21807 |
| *pet*A_CDS | LSC | 90 | 963 | 86670 | 0.65 | 0.049 | 6 | 4 | 26505 | 15574 | 18890 | 25138 |
| *psb*J_CDS | LSC | 90 | 123 | 11070 | 0.208 | 0.008 | 60.2 | 39.8 | 2329 | 1800 | 2605 | 4313 |
| *psb*L_CDS | LSC | 90 | 117 | 10530 | 0.627 | 0.009 | 67.4 | 32.6 | 2956 | 1796 | 1612 | 4100 |
| *psb*F_CDS | LSC | 90 | 120 | 10800 | 0.509 | 0.042 | 57.8 | 42.2 | 2623 | 2390 | 2144 | 3588 |
| *psb*E_CDS | LSC | 90 | 252 | 22680 | 0.049 | 0.016 | 59.5 | 40.5 | 5851 | 4241 | 4932 | 7645 |
| *pet*L_CDS | LSC | 80 | 96 | 7680 | 4.258 | 0.062 | 68.5 | 31.5 | 1936 | 1233 | 1088 | 3096 |
| *pet*G_CDS | LSC | 89 | 114 | 10146 | 1.745 | 0.035 | 6.3 | 3.7 | 2360 | 1558 | 2126 | 3925 |
| *trn*W_CCA_tRNA | LSC | 89 | 74 | 6586 | 0 | 0.014 | 47.6 | 52.4 | 1445 | 1603 | 1848 | 1690 |
| *trn*P_UGG_tRNA | LSC | 90 | 74 | 6660 | 0 | 0.014 | 51.3 | 48.7 | 1436 | 1350 | 1894 | 1980 |
| *psa*J_CDS | LSC | 90 | 135 | 12150 | 1.169 | 0.03 | 57.7 | 42.3 | 2746 | 2677 | 2399 | 4186 |
| *rpl*33_CDS | LSC | 90 | 201 | 18090 | 1.99 | 0.08 | 61.7 | 38.3 | 6533 | 2839 | 3954 | 4404 |
| *rps*18_CDS | LSC | 90 | 311 | 27990 | 5.123 | 0.045 | 65.5 | 34.5 | 9282 | 4446 | 4696 | 8132 |
| *rpl*20_CDS | LSC | 90 | 387 | 34830 | 4.539 | 0.057 | 65.5 | 34.5 | 12401 | 4927 | 6503 | 9418 |
| *rps*12_CDS | LSC | 90 | 898 | 80820 | 0.171 | 0.006 | 59.5 | 40.5 | 25878 | 15992 | 16718 | 22094 |
| *clp*P_CDS_c | LSC | 90 | 1921 | 172890 | 4.812 | 0.073 | 64.1 | 35.9 | 53194 | 26754 | 32317 | 52306 |
| *psb*B_CDS | LSC | 90 | 1527 | 137430 | 0.037 | 0.045 | 55.7 | 44.3 | 32375 | 24842 | 36022 | 44140 |
| *psb*T_CDS | LSC | 87 | 108 | 9396 | 6.705 | 0.074 | 66.3 | 33.7 | 2482 | 1569 | 1390 | 3325 |
| *psb*N_CDS | LSC | 88 | 132 | 11616 | 1.507 | 0.03 | 56.3 | 43.7 | 3110 | 2679 | 2332 | 3320 |
| *psb*H_CDS | LSC | 90 | 222 | 19980 | 1.562 | 0.054 | 61.3 | 38.7 | 5518 | 2998 | 4616 | 6536 |
| *pet*B_CDS_a | LSC | 90 | 1364 | 122760 | 6.858 | 0.065 | 63.5 | 36.5 | 33664 | 18171 | 23496 | 39010 |
| *pet*D_CDS_a | LSC | 90 | 1214 | 109260 | 4.825 | 0.054 | 64.3 | 35.7 | 31461 | 17493 | 19637 | 35397 |
| *rpo*A_CDS | LSC | 90 | 1008 | 90720 | 7.261 | 0.074 | 64.6 | 35.4 | 29318 | 13803 | 15828 | 25184 |
| *rps*11_CDS | LSC | 90 | 417 | 37530 | 0.312 | 0.055 | 54.9 | 45.1 | 11351 | 7428 | 9449 | 9185 |
| *rpl*36_CDS | LSC | 89 | 114 | 10146 | 0.158 | 0.061 | 61.5 | 38.5 | 3901 | 1681 | 2221 | 2327 |
| *inf*A_CDS | LSC | 90 | 234 | 21060 | 1.016 | 0.047 | 62.2 | 37.8 | 7091 | 3056 | 4821 | 5878 |
| *rps*8_CDS | LSC | 90 | 405 | 36450 | 6.368 | 0.069 | 64.8 | 35.2 | 12304 | 5256 | 6729 | 9840 |
| *rpl*14_CDS | LSC | 90 | 369 | 33210 | 1.298 | 0.054 | 61.2 | 38.8 | 10960 | 5552 | 7163 | 9104 |
| *rpl*16_CDS_a | LSC | 90 | 1363 | 122670 | 12.636 | 0.09 | 64.1 | 35.9 | 39829 | 16717 | 21723 | 28900 |
| *rps*3_CDS | LSC | 90 | 663 | 59670 | 6.082 | 0.078 | 65.1 | 34.9 | 21793 | 8921 | 10628 | 14699 |
| *rpl*22_CDS | LSC | 88 | 468 | 41184 | 1.556 | 0.096 | 64.6 | 35.4 | 15155 | 6456 | 7876 | 11056 |
| *rps*19_CDS | LSC | 74 | 36 | 2664 | 0.938 | 0 | 69.6 | 30.4 | 1024 | 436 | 367 | 812 |
| *rpl23*_CDS | IR-B | 90 | 282 | 25380 | 0.02 | 0.011 | 62.1 | 37.9 | 8911 | 4226 | 5397 | 6841 |
| *trn*I_CAU_tRNA | IR-B | 90 | 74 | 6660 | 0 | 0 | 55.4 | 44.6 | 1890 | 1530 | 1440 | 1800 |
| *ycf*2_CDS | IR-B | 90 | 6870 | 618300 | 0.746 | 0.016 | 61.9 | 38.1 | 190433 | 114234 | 119317 | 189703 |
| *ycf*15_CDS | IR-B | 90 | 249 | 22410 | 1.151 | 0.028 | 58.6 | 41.4 | 7302 | 4356 | 4795 | 5699 |
| *trn*L_CAA_tRNA | IR-B | 90 | 81 | 7290 | 0 | 0 | 48.1 | 51.9 | 1890 | 1530 | 2250 | 1620 |
| *ndh*B_intron | IR-B | 90 | 2208 | 198720 | 0.263 | 0.006 | 62.1 | 37.9 | 57526 | 38247 | 36782 | 65643 |
| *rps*7_CDS | IR-B | 90 | 468 | 42120 | 0 | 0.006 | 60.3 | 39.7 | 15209 | 8099 | 8646 | 10166 |
| *trn*V_GAC_tRNA | IR-B | 90 | 72 | 6480 | 0 | 0 | 51.4 | 48.6 | 1620 | 1350 | 1800 | 1710 |
| *rrn*16S rRNA | IR-B | 90 | 1491 | 134190 | 0 | 0.001 | 43.5 | 56.5 | 33108 | 32309 | 43480 | 25293 |
| *trn*I_GAU_tRNA_a | IR-B | 90 | 1012 | 91080 | 0.019 | 0.005 | 49.7 | 50.3 | 21649 | 21633 | 24198 | 23583 |
| *trn*A_UGC_tRNA_a | IR-B | 90 | 868 | 78120 | 0.01 | 0.01 | 48.6 | 51.4 | 19181 | 17126 | 22994 | 18811 |
| *rrn*23S_rRNA | IR-B | 90 | 2811 | 252990 | 0.006 | 0.006 | 45.2 | 54.8 | 67394 | 59502 | 79196 | 46883 |
| rrn4.5S_rRNA | IR-B | 90 | 103 | 9270 | 0 | 0 | 49.5 | 50.5 | 2610 | 1799 | 2880 | 1981 |
| *rrn*5S_rRNA | IR-B | 90 | 121 | 10890 | 0 | 0 | 47.9 | 52.1 | 2790 | 2610 | 3060 | 2430 |
| *trn*R_ACG_tRNA | IR-B | 90 | 74 | 6660 | 0.015 | 0 | 37.8 | 62.2 | 1259 | 1980 | 2160 | 1260 |
| *trn*N_GUU_tRNA | IR-B | 90 | 72 | 6480 | 0 | 0 | 45.8 | 54.2 | 1169 | 1350 | 2161 | 1800 |
| *ycf*1_CDS | IR-B | 88 | 6042 | 531696 | 24.915 | 0.106 | 68.2 | 31.8 | 153698 | 61840 | 64152 | 119532 |
| *ndh*F_CDS | SSC | 90 | 2233 | 200970 | 8.44 | 0.106 | 66.7 | 33.3 | 49533 | 28530 | 32629 | 73317 |
| *rpl*32_CDS | SSC | 87 | 171 | 14877 | 7.784 | 0.053 | 66.5 | 33.5 | 5193 | 1952 | 2619 | 3955 |
| *trn*L_UAG_tRNA | SSC | 90 | 80 | 7200 | 0.194 | 0.025 | 44.9 | 55.1 | 1608 | 1618 | 2342 | 1618 |
| *ccs*A_CDS | SSC | 90 | 978 | 88020 | 11.344 | 0.075 | 69.6 | 30.4 | 24819 | 11317 | 12206 | 29693 |
| *ndh*D_CDS | SSC | 90 | 1529 | 137610 | 3.966 | 0.08 | 63.9 | 36.1 | 34542 | 23869 | 23845 | 49896 |
| *psa*C_CDS | SSC | 90 | 246 | 22140 | 0.172 | 0.049 | 56.8 | 43.2 | 5937 | 3880 | 5668 | 6617 |
| *ndh*E_CDS | SSC | 87 | 306 | 26622 | 6.987 | 0.039 | 69.2 | 30.8 | 7528 | 3768 | 3886 | 9580 |
| *ndh*G_CDS | SSC | 88 | 531 | 46728 | 5.964 | 0.055 | 66.4 | 33.6 | 11776 | 7239 | 7507 | 17419 |
| *ndh*I_CDS | SSC | 90 | 507 | 45630 | 5.483 | 0.055 | 64.8 | 35.2 | 13109 | 7645 | 7474 | 14900 |
| *ndh*A_intron | SSC | 90 | 2097 | 188730 | 11.383 | 0.084 | 67.3 | 32.7 | 53333 | 24879 | 29593 | 59441 |
| *ndh*H_CDS | SSC | 90 | 1182 | 106380 | 0.852 | 0.061 | 61.2 | 38.8 | 32107 | 16598 | 24304 | 32465 |
| *rps*15_CDS | SSC | 88 | 273 | 24024 | 5.644 | 0.092 | 67.2 | 32.8 | 8939 | 3164 | 4274 | 6291 |
| *rpl*2_CDS | IR-A | 90 | 1483 | 133470 | 0.131 | 0.008 | 58 | 4.2 | 44471 | 24098 | 31908 | 32818 |
| **Average** |  | 89.2 | 811.1 (Total 92461) | 72690 | 2.6462 | 0.04079 | 54.27 | 41.7 | 20882.83 | 12436.54 | 14700.43 | 21142.28 |
