# Supplementary material for: Phylogenomics of Salvia L. subgenus Calosphace (Lamiaceae)
Source: Front Plant Sci. 2021 Oct 15;12:725900. doi: 10.3389/fpls.2021.725900 (PMC8554000; doi:10.3389/fpls.2021.725900)

Supplemental Material Figures Hyb-seq *Salvia*

Supplementary Figure 1. ASTRAL analysis 96 nuclear genes, local PP is indicated above branches >0.7 (lower are collapsed). *Salvia* subgenus *Calosphace* sections *s*. Epling are color coded. Main clades are indicated following previous nomenclature (Walker et al. 2004, Jenks et al. 2013, Fragoso-Martinez et al. 2018).


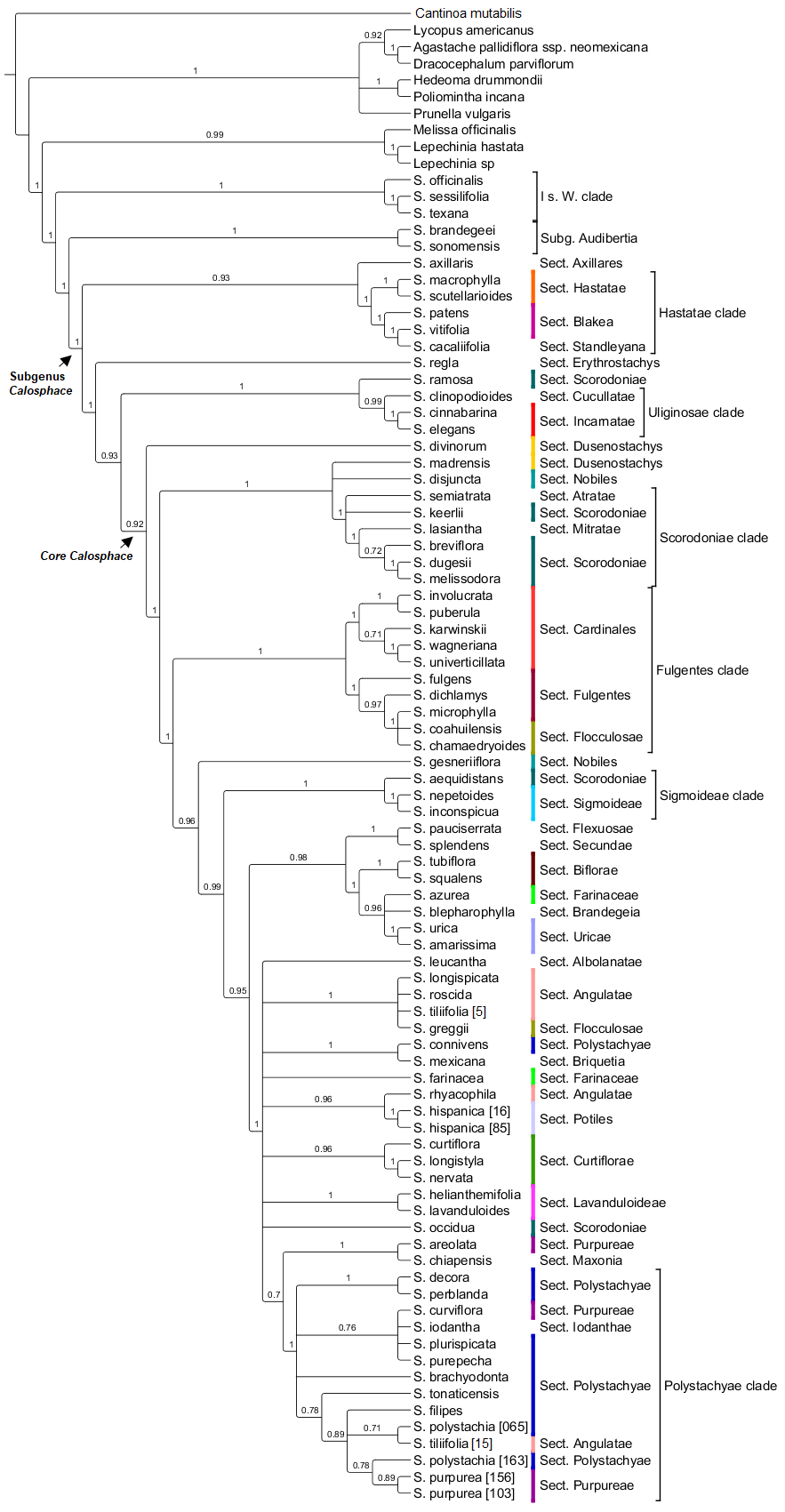


Supplementary Figure 2. Nuclear ASTRAL trees under distinct missing data stringencies; A (30% missing), B (50% missing), C (75% missing).


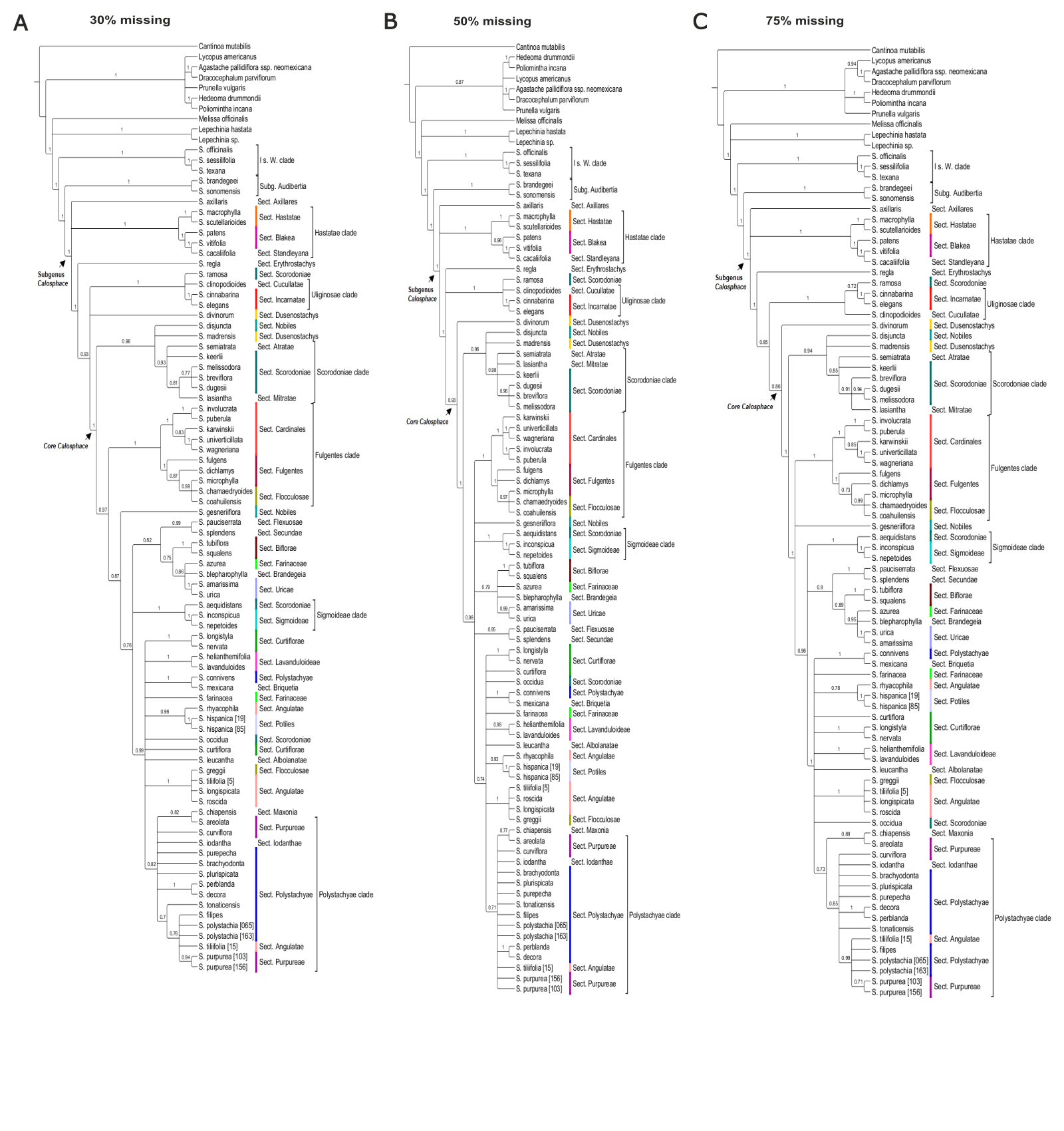


Supplementary Figure 3. Chloroplast ASTRAL trees under distinct missing data stringencies; A (30% missing), B (50% missing), C (75% missing).


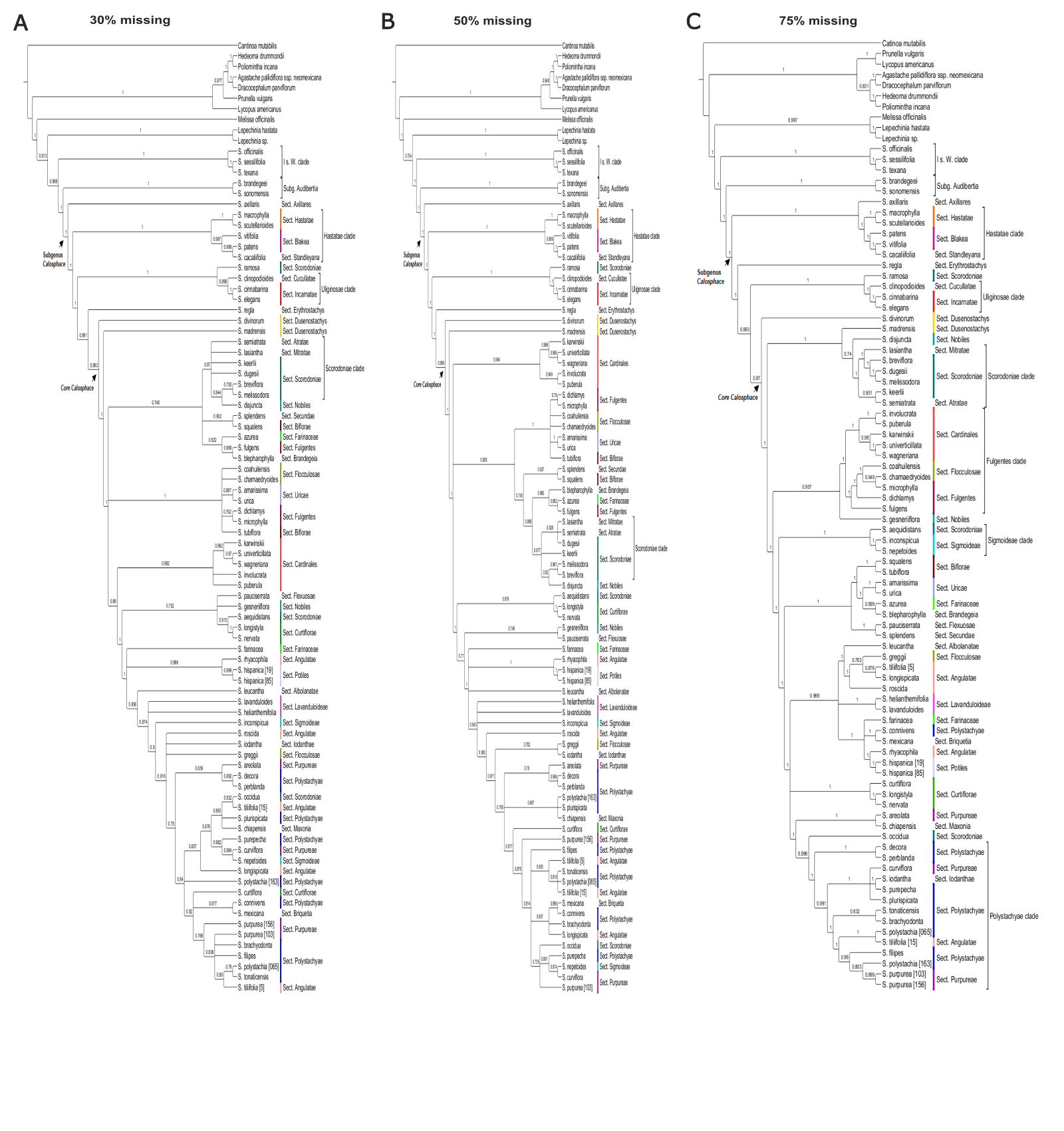

Supplement: Supplementary file 6 [file Data_Sheet_1.docx]
